# Secure Obfuscation for Encrypted Group Signatures

**DOI:** 10.1371/journal.pone.0131550

**Published:** 2015-07-13

**Authors:** Yang Shi, Qinpei Zhao, Hongfei Fan, Qin Liu

**Affiliations:** School of software engineering, Tongji University, Shanghai, China; Mathematical Institute, HUNGARY

## Abstract

In recent years, group signature techniques are widely used in constructing privacy-preserving security schemes for various information systems. However, conventional techniques keep the schemes secure only in normal black-box attack contexts. In other words, these schemes suppose that (the implementation of) the group signature generation algorithm is running in a platform that is perfectly protected from various intrusions and attacks. As a complementary to existing studies, how to generate group signatures securely in a more austere security context, such as a white-box attack context, is studied in this paper. We use obfuscation as an approach to acquire a higher level of security. Concretely, we introduce a special group signature functionality-an encrypted group signature, and then provide an obfuscator for the proposed functionality. A series of new security notions for both the functionality and its obfuscator has been introduced. The most important one is the average-case secure virtual black-box property w.r.t. dependent oracles and restricted dependent oracles which captures the requirement of protecting the output of the proposed obfuscator against collision attacks from group members. The security notions fit for many other specialized obfuscators, such as obfuscators for identity-based signatures, threshold signatures and key-insulated signatures. Finally, the correctness and security of the proposed obfuscator have been proven. Thereby, the obfuscated encrypted group signature functionality can be applied to variants of privacy-preserving security schemes and enhance the security level of these schemes.

## Introduction

Group signature was proposed by Cham and Heyst [1], which is a special type of digital signature for a group of persons. In the group signature setting, there is a group having numerous members and a single manager (the group manager). A single verification key called the group public key is associated with the group. Each group member has its own secret signing key based on which it can produce a signature relative to the group public key. The group manager has a master secret key. Given a signature Σ, based on the master secret key, the group manager can extract the identity of the group member who created Σ. It is called traceability. On the other hand, those who are not holding the master secret key are unable to extract the identity of the group member who created Σ, which is called anonymity. In fact, a group signature has the following properties[1,2]: (i) only members of the group can sign messages; (ii) the receiver can verify whether it is a valid group signature; (iii) anonymity; (iv) traceability.

Group signature schemes provide functionalities which are applicable in many practical scenarios. In recent years, applications of group signature schemes in many emerging technologies or privacy-sensitive applications are studied, such as in social networks [3,4], medical information systems [5–7], Vehicular Ad hoc Networks (VANets) [8,9], electronic voting [10], Wireless Sensor Networks (WSNs) [11], electronic cash [12,13], and cloud computing [14–18] especially. These studies make great contributions for protecting security of information systems and privacy of users against various attacks. However, all these schemes are developed in the black-box model (or the so-called “black-box attack context”). In a black-box attack context, an adversary can access only the functionality of a cryptosystem. However, there is another security model (the white-box model) in which an adversary has total visibility of the implementation of the cryptosystem and full control over its execution platform. That is, the implementation of the cryptosystem is running in a White-Box Attack Context (WBAC).

In fact, we can find many typical WBACs, such as (1) a server or an endpoint for which a hacker has got the “root” or “admin” privilege; (2) a malicious host where mobile agents are running [19,20]; (3) an outdoor sensor node (of a WSN) captured by an attacker [21,22]; (4) the Digital Right Management (DRM) components in cable TV set-top boxes or IPTV equipments [23,24]; (5) On Board Units (OBUs) and Road Side Units (RSUs) in a VANet (e.g., the device suffers from the so called "Industrial insiders’ attack" in [25] or the "On-board tampering" attack in [26], or even the “Malware attack” in [27]); and (6) mobile devices (e.g., smart phones and tablets) captured by an attacker [28].

In WBACs, obfuscating techniques should be used while implementing key-related cryptographic functionalities to protect privacy-preserving schemes or security protocols based on group signature schemes. Hence, we contribute to the security requirements as follows.

1. A special obfuscatable group signature functionality, i.e., the encrypted group signature, is proposed with a concrete scheme, and then a corresponding obfuscator is provided.
2. Security notions of the encrypted group signature functionality and notions of the corresponding obfuscators are proposed. The most important one of the new security notions is average-case secure virtual black-box property (ACVBP) w.r.t. Dependent Oracles and Restricted Dependent Oracles, which describes the security requirement of protecting the output of the proposed obfuscator, i.e., the obfuscated implementation of encrypted group signature functionality against collision attacks from group members. The security notions fit for many other application scenarios.
3. The correctness and security of the proposed obfuscator are proven. The efficiency of the proposed encrypted group signature functionality and its obfuscator is analyzed.

Besides the contributions to cryptography, the result is useful in many applications. For example, the proposed technique can be applied in cloud computing. In this application, an inner user can upload a file anonymously on the private cloud of a company. However, in the case that an investigation is needed, the group manager is capable of opening the identity of the user. For another example, in a privacy-preserving emergency call (PEC) scheme for mobile healthcare social networks, the obfuscatable encrypted group signature scheme and its obfuscator can be used to implement a decentralized emergency response system for a rapid response of emergency care in the network. The application demonstrates that, with the help of encrypted group signature technique, the PEC preserves users’ privacy by hiding their identities, and it avoids unnecessary disclosure of personal health information. The details of these applications are provided in Section 6.1.1 and 6.1.2 to demonstrate the applicability in concrete scenarios.

Furthermore, we found that the proposed solution can be adapted to identity-based cryptography and key-insulated cryptography. Therefore, how to transform the proposed obfuscatable encrypted group signature scheme into an obfuscatable encrypted identity-based signature scheme and an obfuscatable encrypted key-insulated signature scheme are sketched out in Section 6.1.3.

The remainder of this paper is organized as follows. The next section presents background information about obfuscation and obfuscators. The section also provides a brief introduction on the complexity assumptions needed in this paper. Section 3 first proposes an overall scheme that is a combination of a group signature scheme and an asymmetric encryption scheme, then an obfuscatable Encrypted Group Signature (EGS) functionality based on the overall scheme is provided. Section 4 presents an obfuscator for the proposed EGS functionality and proves the correctness and security of the obfuscator. Moreover, a series of security notions of the functionality and the obfuscators is also introduced in Section 4. In Section 5, we compare the results of the proposed scheme with the ones in other studies on obfuscation for cryptographic purpose. A discussion on possible applications and extensions of the proposed scheme and the obfuscator is provided in Section 6. The rationale behind the obfuscatable sign-then-encrypt functionalities and our main contributions is also discussed in this section. Finally, the article concludes in Section 7 with future work.

## Preliminaries and Background

### 2.1 Obfuscation and Its Recent Advances

Informally, the goal of obfuscation is to make a computer program "unintelligible" while preserving its functionality, and an obfuscator is a “compiler” that performs such transformations [29,30]. As the results in [29–33] are mainly about the difficulties or even impossibilities of obfuscation, it is a hard work to find a secure obfuscator, even though for a special functionality. Some positive results were reported besides these negative results for general-purpose obfuscation, such as in [29–33]. However, these positive results mainly serve as theoretical illustrations and many of these positive results are not suitable for practical applications.

Despite these positive results for theoretical study, obfuscators for cryptographic-related functionalities with acceptable runtime cost remained elusive until the obfuscatable encrypted signature [34] and the obfuscatable re-encryption [35] were proposed. Fortunately, after these two articles were published, several obfuscatable cryptographic-related functionalities were identified and the corresponding secure obfuscators were proposed in recent years [36–41].

We note that these positive results do not violate the general impossibility results. One of the reasons is that they are not general-purpose obfuscation. The other one is that they have used distinct weak security criteria such as the ACVBP proposed by Hohenbergeret al. [35] or the simulation-based average-case virtual black-box property proposed by Hofheinz et al. [42].

Two general-purpose obfuscators [43,44] were proposed in recent two years, however, they either use a weak security notion or use the homomorphic encryption techniques with high costs of space and time.

Both general-purpose obfuscators and specialized obfuscators for cryptographic functionalities usually set the input as a (probabilistic) circuit in theoretical analysis. A brief review on probabilistic circuits and circuit obfuscators is provided in the following subsection.

### 2.2. Probabilistic Circuits and Circuit Obfuscators

As prerequisites of security analysis, we use the conventional definitions and notations of probabilistic circuits and circuit obfuscators following those in [29,30,34,35].

Let Poly(*λ*) denote the set of all polynomials of *λ*. Let ℂ_*λ*_ be a set of polynomial-size circuits with input length *l*_*Input*_(*λ*) ∈ Poly(*λ*) and output length *l*_*Output*_(*λ*) ∈ Poly(*λ*). Usually, we use ℂ = {ℂ_*λ*_}_*λ*∈ℕ_ to denote a class of such circuits, where there exists an associated Probabilistic Polynomial Time (PPT) generation algorithm which takes as input 1^*λ*^ and generates a random circuit ℂ ∈_$_ ℂ_*λ*_. In studies on obfuscation for cryptographic purpose, it usually corresponds to the random generation of system parameters or cryptographic keys on the security parameter 1^*λ*^. The generation process is denoted by *C* ← ℂ_*λ*_. When a circuit *C* is used as an input argument or an output result of an algorithm, it is assumed that a specification of the circuit is used implicitly.

Let *para* be the set of regular input parameters and *rand* be the random input variable. Suppose that *C(para*, *rand)* is a probabilistic circuit. Given an arbitrary regular input *para*, we can consider *C*(*para*,·) (or *C*_*para*_(·)) as a sampling algorithm for the distribution obtained by evaluating the output of *C(para*, *rand)* on random variable *rand*. Given a couple of probabilistic circuits (*C*_0_, *C*_1_) whose regular inputs are of the same length, the two distributions produced by *C*_0_(*para*,·) and *C*_1_(*para*,·) are denoted by the pair (*C*_0_(*para*), *C*_1_(*para*)). The statistical difference between them is defined in (1) [34].

$$\begin{array}{l} \text{StaDiff}\left(C_{0}\left(para\right),\;C_{1}\left(para\right)\right)\\ =\frac{1}{2}\sum_{y\in {\left\{0,\text{1}\right\}}^{l_{Output}(\lambda )}}\left|\text{Pr}[out\leftarrow C_{0}(para)\;:out=y]-\text{Pr}[out\leftarrow C_{1}(para)\;:out=y]\right| \end{array}$$ (1)

Moreover, for a Turing machine *M*, its black-box access to a probabilistic circuit *C* can be divided into two categories, i.e., “oracle access” and “sampling access”. Oracle access means that the Turing machine *M* is allowed to set both regular and random inputs. It is denoted by *M* ^*C*^ as the traditional way. Sampling access means that the Turing machine *M* is only allowed to set the regular input. It is denoted by *M* ^≪*C≫*^.

An obfuscator for a class of circuits ℂ = {ℂ_*λ*_}_*λ*∈ℕ_ is a PPT machine which takes a circuit *C* ∈ ℂ_*λ*_ as input and outputs an unintelligible circuit *C'* which preserves the functionality. The formal description of “preserving functionality” is given by Definition 1 in section 4.2.

### 2.3. Complexity Assumptions

The overall scheme containing the obfuscatable EGS functionality in this paper is built based on a group signature scheme [45] and an asymmetric linear encryption scheme [46], so, we make use of the following four complexity assumptions as the theoretical basis of our work.

#### Remark 1

Although most cryptosystems based on pairings assume for simplicity that bilinear groups have prime order. In our case, it is important that the pairing is defined over a group *G* containing |*G*| = *n* elements, where *n* = *pq* has a (ostensibly hidden) factorization in two large primes, *p* ≠ *q*. Moreover, *G*_*p*_ and *G*_*q*_ denote the cyclic subgroups of *G* with respective order *p* and *q*.

- The Computational Diffie-Hellman (CDH) assumption in bilinear groups. This assumption states that, given a triple $(g,\;g^{a},\;g^{b})\in G_{p}^{3}$ for random exponents *a*, *b* ∈ Z_*p*_, there is no PPT algorithm that can compute *g* ^*ab*^ ∈ *G*_*p*_ with non-negligible probability. Because of the bilinear pairing, CDH in *G*_*p*_ implies a “Gap Diffie Hellman” assumption.
- The subgroup decision assumption. Consider an “instance generator” algorithm *𝓖𝓖* that, on input a security parameter 1^*λ*^, outputs a tuple (*p*, *q*, *G*, *G*_*T*_, *e*), in which *p* and *q* are independent uniform random *λ*-bit primes, *G* and *G*_*T*_ are cyclic groups of order *n* = *pq* with efficiently computable group operations (over their respective elements, which must have a polynomial size representation in *λ*), and *e*: *G* × *G → G*_*T*_ is a bilinear map. The subgroup decision assumption is that on input a tuple (*n* = *pq*, *G*, *G*_*T*_, *e*) derived from a random execution of *𝓖𝓖*(1^*λ*^), and an element *w* selected at random either from *G* or from *G*_*q*_, there is no (PPT) algorithm can decide whether *w* ∈ *G*_*q*_ with non-negligible advantage.
- The Hidden Strong Diffie-Hellman assumption. We first define the *l*-HSDH problem as follows: On input three generators *g*, *h* and *g* ^*ω*^ of *G*_*p*_, and *l*-1 distinct triples $\left(g^{1/\left(\omega +c_{i}\right)},\;g^{c_{i}},\;h^{c_{i}}\right)\in G_{p}^{3}$ where *c*_*i*_ ∈ Z_*p*_, output another such triple $\left(g^{1/\left(\omega +c\right)},\;g^{c},\;h^{c}\right)\in G_{p}^{3}$ distinct of all the others. The Hidden Strong Diffie-Hellman assumption states that, in a family of prime order bilinear groups generated by the instance generator *𝓖𝓖*, there is no PPT algorithm can solve the HSDH problem in the bilinear group $\left(p,G_{p},\;\hat{e}\right)\leftarrow 𝓖𝓖(1^{\lambda })$ with non-negligible probability for sufficiently large *λ* ∈ ℕ.
- The Decisional Linear (DLIN) assumption. We first define the Decision Linear Problem in *G* as follows: Given *u*, *v*, *h*, *u* ^*a*^, *v* ^*b*^, *h* ^*c*^ ∈ *G* as input, output yes if *a*+*b* = *c* and no otherwise. The DLIN assumption states that, there is no (PPT) algorithm can solve the Decision Linear Problem with non-negligible advantage.

#### Remark 2

The DLIN problem is originally defined in a prime-order group in [46]. In our case, we use the DLIN assumption over composite-order group, similar assumptions could be found in literatures such as [47] and [48].

## An Obfuscatable Encrypted Group Signature Functionality

We propose an overall scheme that is a combination of a group signature scheme and an asymmetric encryption scheme. The scheme consists of seven algorithms: Setup, Enroll, Sign, Verify, EKGen, Enc and Dec. Based on the scheme, an obfuscatable EGS algorithm implements the EGS functionality is then provided.

### 3.1. The Overall Scheme

**Fig 1:**
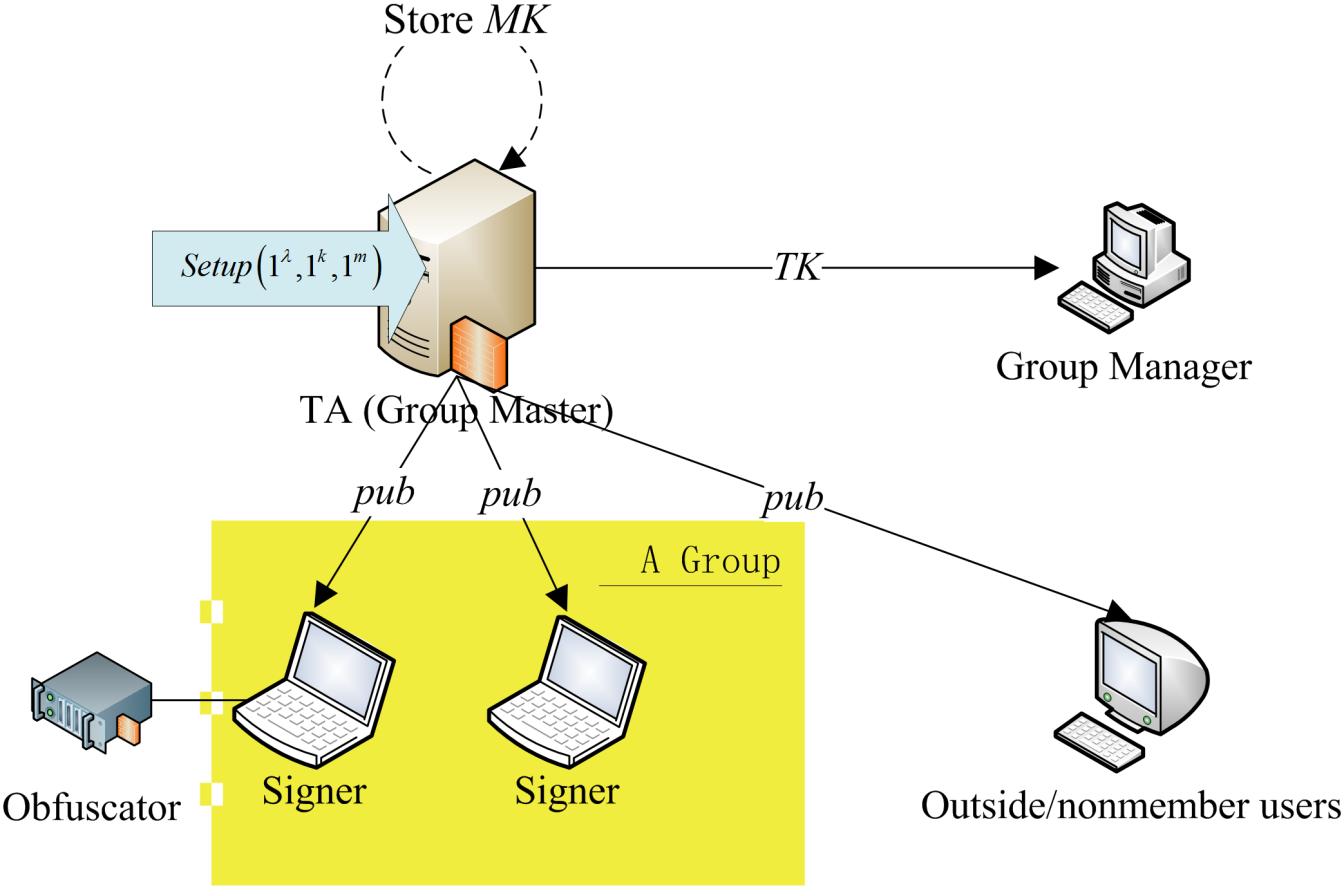
The usage of the Setup algorithm.

There are seven types of roles (see Fig 1) in the scheme: The first type is the group master. It is a trusted authority (TA) which is in charge of initializing system parameters, generating public parameters, setting up the group and issuing secret signing keys to the group members. Furthermore, the TA also certificates public encryption keys for all users (member or nonmember of the group). Sometimes, the master key *MK* is destroyed once the group is set up. The second type is the group manager, which is given the ability to identify signers using the tracing key *TK*, but not to enroll new users or create new signing keys. The third type is regular member users (group members, or signers) that each one is given a distinct secret signing key *K*_ID_. The fourth type is verifiers, who can verify signatures using the public parameters. The fifth type is encryptors and the sixth type is decryptors which are not shown in Fig 1 as they are too simple. These three types (i.e., the fourth type, the fifth type and the sixth type) of users could be either a group member of nonmember. The seventh type is obfuscators.

Algorithms proposed in [45] (a group signature scheme) and [46] (an asymmetric linear encryption scheme) are used to construct the overall scheme. Hence, the algorithms of the overall scheme are similar to the corresponding algorithms in [45] and [46]; in fact, they are slightly modified or only different in description. Note that it is not a trivial work to the overall scheme because the signing algorithm Sign and the encryption algorithm Enc should be “compatible” to construct the obfuscatable EGS functionality. These algorithms in the scheme are introduced in the following subsections. To make the algorithms easier to understand, we list frequently used symbols in Table 1.

**Table 1:** Symbols.

Symbol	Description
*pub*	A tuple consists of system parameters and public values
*MK*	The master enrollment key
*TK*	The group manager’s tracing key
2^*k*^	The maximum number of signers
{0,1}^*m*^	The message space
*G*, *G*_*T*_	Cyclic groups
*G*_*p*_, *G*_*q*_	Subgroups of *G*
ID	The user’s identity
*s*_ID_	A secret unique value corresponding to ID
*K*_ID_	The private signing key
*M*	A message
*σ*	A signature
*PK*_*e*_	The encryption key
*SK*_*e*_	The decryption key
*C*	Ciphertext

#### 3.1.1. Setup

The Setup algorithm takes a security parameter in unary as input. The algorithm outputs *pub* (a tuple consists of system parameters and public values), the master enrollment key *MK*, and the group manager’s tracing key *TK*. Suppose that up to 2^*k*^ signers are supported in the group, and the message space is {0,1}^*m*^, where *k* = *O*(*λ*) and *m* = *O*(*λ*). Let *Gen*[*group*] denote the set of generators of the “*group*”. The usage of the algorithm is illustrated in Fig 1.

The algorithm proceeds as follows.

**Algorithm** *Setup*(1^λ^,1^*k*^,1^*m*^)

Begin

$p,q\overset{\$}{\leftarrow }\mathbb{N}$,*p* and *q* are prime numbers s.t. log_2_ *p* = Θ(λ)>*k*∧log_2_q=Θ(λ)>*k*

*n←p·q*

*builss a cyclic bilinear group G* of order n

 Let *G*_*p*_ be the cyclic subgroups of *G* of order *p*

 Let *G*_*q*_ be the cyclic subgroups of *G* of order *q*

$h\overset{\$}{\leftarrow }Gen\left[G_{q}\right]$

$g\overset{\$}{\leftarrow }Gen\left[G\right]$

$\alpha ,\omega \overset{\$}{\leftarrow }Z_{n}$

$A\leftarrow \hat{e}(g,\;g)\in G_{T}$

 Ω←*g* ^ω^ ∈ *G*

*u*,*v*',*v*_1_,*…*,*v*_*m*_ ∈ *Gen*[*G*]

*PP*←(*g*,*h*,*u*,*v*’,*v*_1_,*…*,*v*_*m*,_Ω,*A*) ∈ *G × G*_*q*_ × *G* ^*m+3*^ × *G*_*T*_

$pub\leftarrow \left(n,\hat{e},G,G_{T},PP\right)$

*MK*←(*g* ^*a*^,*ω*) ∈ *G* × *Z*_*n*_

*TK→q* ∈ ℕ

*output* (*pub*,*MK*,*TK*)

End

#### 3.1.2. Enroll

**Fig 2:**
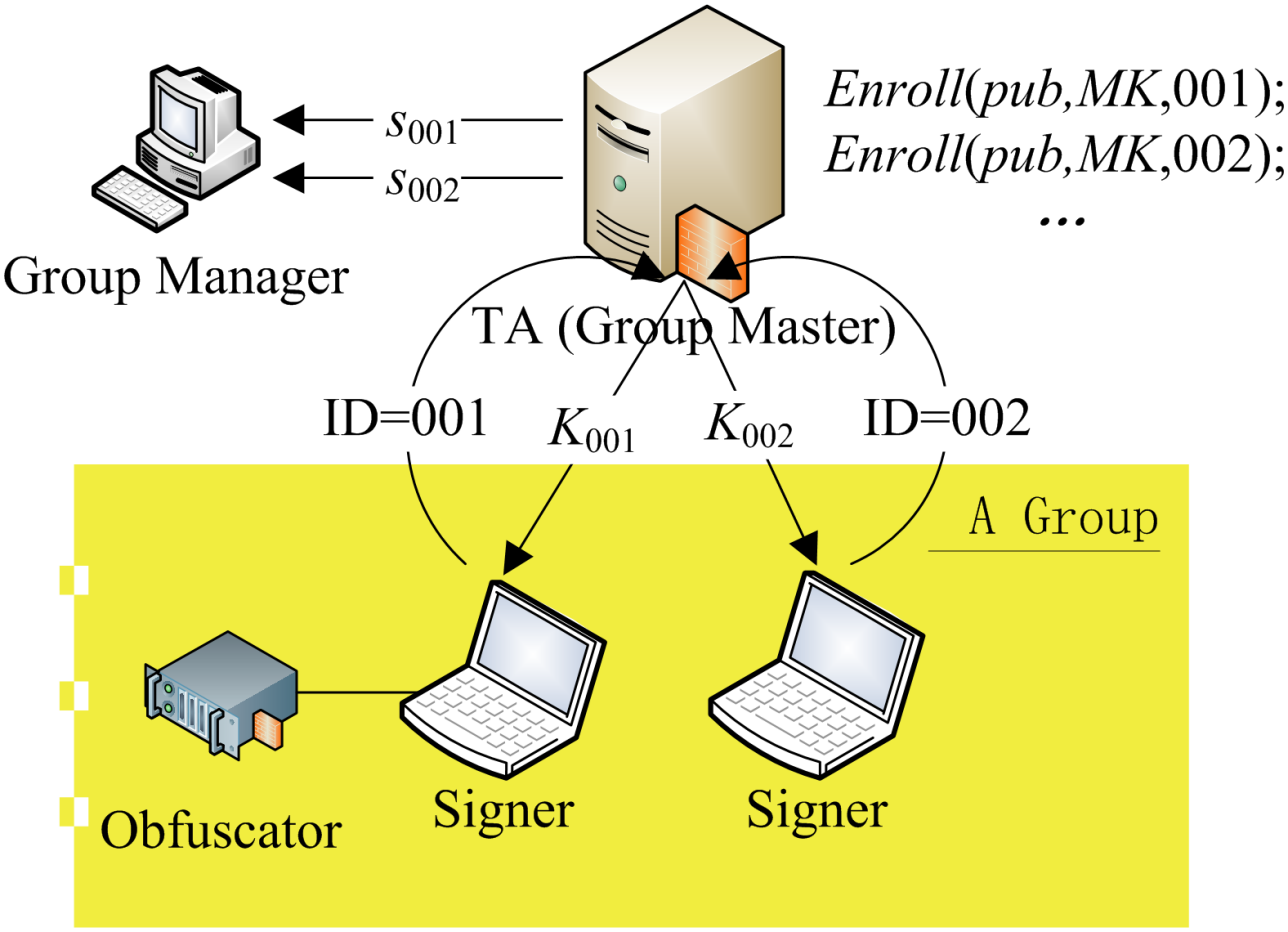
The usage of the Enroll algorithm.

The algorithm Enroll serves for creating a signing key for a user whose identity is ID, where 0≤ID< 2^*k*^ <*p*. The enroll algorithm takes *pub* (a tuple consists of system parameters and public values), the master key *MK*, and the user’s identity ID as input. It outputs a unique identifier *s*_ID_ ∈ Z_*n*_ and a corresponding private signing key *K*_ID_. The secret unique value *s*_ID_ can be later used for tracing purposes. This value must be chosen so that *ω*+*s*_ID_ lies in $Z_{n}^{*}$. The usage of the algorithm is illustrated in Fig 2.

The algorithm proceeds as follows.

**Algorithm** *Enroll* (*pub*,*MK*, ID)

Begin

$\left(n,\hat{e},G,G_{T},PP\right)\leftarrow pub$

 (*g*, *h*, *u*, *v*', *v*_1_,…,*v*_*m*_, Ω, *A*) ← *PP*

 (*g* ^*α*^,*ω*) ← *MK*

$s_{\text{ID}}\in_{\$}Z_{n},s.t._{}\omega +s_{\text{ID}}\in Z_{n}^{*}$

$K_{\text{ID}}=\;(K_{\text{1}},K_{\text{2}},K_{\text{3}})\leftarrow \left({\left(g^{\alpha }\right)}^{\frac{1}{\omega +s_{\text{ID}}}},\;g^{s_{\text{ID}}},\;u^{s_{\text{ID}}}\right)\in G^{\text{3}}$

End

#### 3.1.3. Sign

The signing algorithm takes *pub*(a tuple consists of system parameters and public values), a group member’s private signing key *K*_ID_, and the message *M* = (*μ*_1_,…, *μ*_*m*_) ∈ {0,1}^*m*^ as input. The algorithm outputs a signature (*σ*_1_, *σ*_2_, *σ*_3_, *σ*_4_, *π*_1_, *π*_2_) using the following procedure.

**Algorithm** *Sign*(*pub, K_ID,_ M*)

Begin

$\left(n,\hat{e},G,G_{T},PP\right)\leftarrow pub$

 (*g*, *h*, *u*, *v'*, *v*_*1*_,..., *v*_*m*_ , Ω, *A*) ← *PP*

$s\overset{\$}{\leftarrow }Z_{n}$

 (*μ*_*1*_,...,*μ*_*m*_) ← *M*

$\theta =(\theta_{\text{1}},\theta_{\text{2}},\theta_{\text{3}},\theta_{\text{4}})=\left(K_{\text{1}},\;K_{\text{2}},\;K_{\text{3}}\cdot {\left(v'\overset{m}{\underset{i=1}{\prod }}v_{i}^{\mu_{i}}\right)}^{s},g^{-s}\right)$

$t_{1},t_{2},t_{3},t_{4}\overset{\$}{\leftarrow }Z_{n}$

$\sigma_{1}\leftarrow \theta_{1}\cdot h^{t_{1}}$

$\sigma_{2}\leftarrow \theta_{2}\cdot h^{t_{2}}$

$\sigma_{3}\leftarrow \theta_{3}\cdot h^{t_{3}}$

$\sigma_{4}\leftarrow \theta_{4}\cdot h^{t_{4}}$

$\pi_{1}\leftarrow h^{t_{1}t_{2}}\cdot {\left(\theta_{1}\right)}^{t_{2}}\cdot {\left(\theta_{2}\Omega \right)}^{t_{1}}$

$\pi_{2}\leftarrow u^{t_{2}}\cdot g^{-t_{3}}\cdot {\left(v'\overset{m}{\underset{i=1}{\prod }}v_{i}^{\mu_{i}}\right)}^{t_{4}}$

*output* (*σ*_1_, *σ*_2_, *σ*_3_, *σ*_4_, *π*_1_, *π*_2_)

End

#### 3.1.4. Verify

The group signature verification algorithm Verify takes *pub*(a tuple consists of system parameters and public values), a signature *σ* from an unknown group member, and a message *M* as input. If the signature *σ* is valid, it outputs 1, otherwise, outputs 0. Note that the verification algorithm outputs 1 only implies that the signature is generated by a member of the given group, but does not reveal the identity of the original signer.

**Algorithm** *Verify*(*pub*, *σ*, *M*)

Begin

 (*σ*_1_, *σ*_2_, *σ*_3_, *σ*_4_, *π*_1_, *π*_2_) ← *σ*

$\left(n,\hat{e},G,G_{T},PP\right)\leftarrow pub$

 (*g*, *h*, *u*, *v*', *v*_1_, …, *v*_*m*_, Ω, *A*) ← *PP*

 (*μ*_1_, …, *μ*_*m*_) ← *M*

$T_{\text{1}}\leftarrow A^{-\text{1}}\cdot \hat{e}(\sigma_{\text{1}},\;\sigma_{\text{2}}\Omega )$

$T_{\text{2}}\leftarrow \hat{e}(\sigma_{\text{2}},\;u)\cdot \hat{e}{\left(\sigma_{\text{3}},\;g\right)}^{-\text{1}}\cdot \hat{e}{\left(\sigma_{\text{4}},\;v'\overset{m}{\underset{i=1}{\prod }}v_{i}{}^{\mu_{i}}\right)}^{-1}$

$\text{IF}\left(T_{1}=\hat{e}(h,\pi_{1})\wedge T_{2}=\hat{e}(h,\pi_{2})\right)$

*output* 1

 Else

*output* 0

End

#### 3.1.5. Open

**Fig 3:**
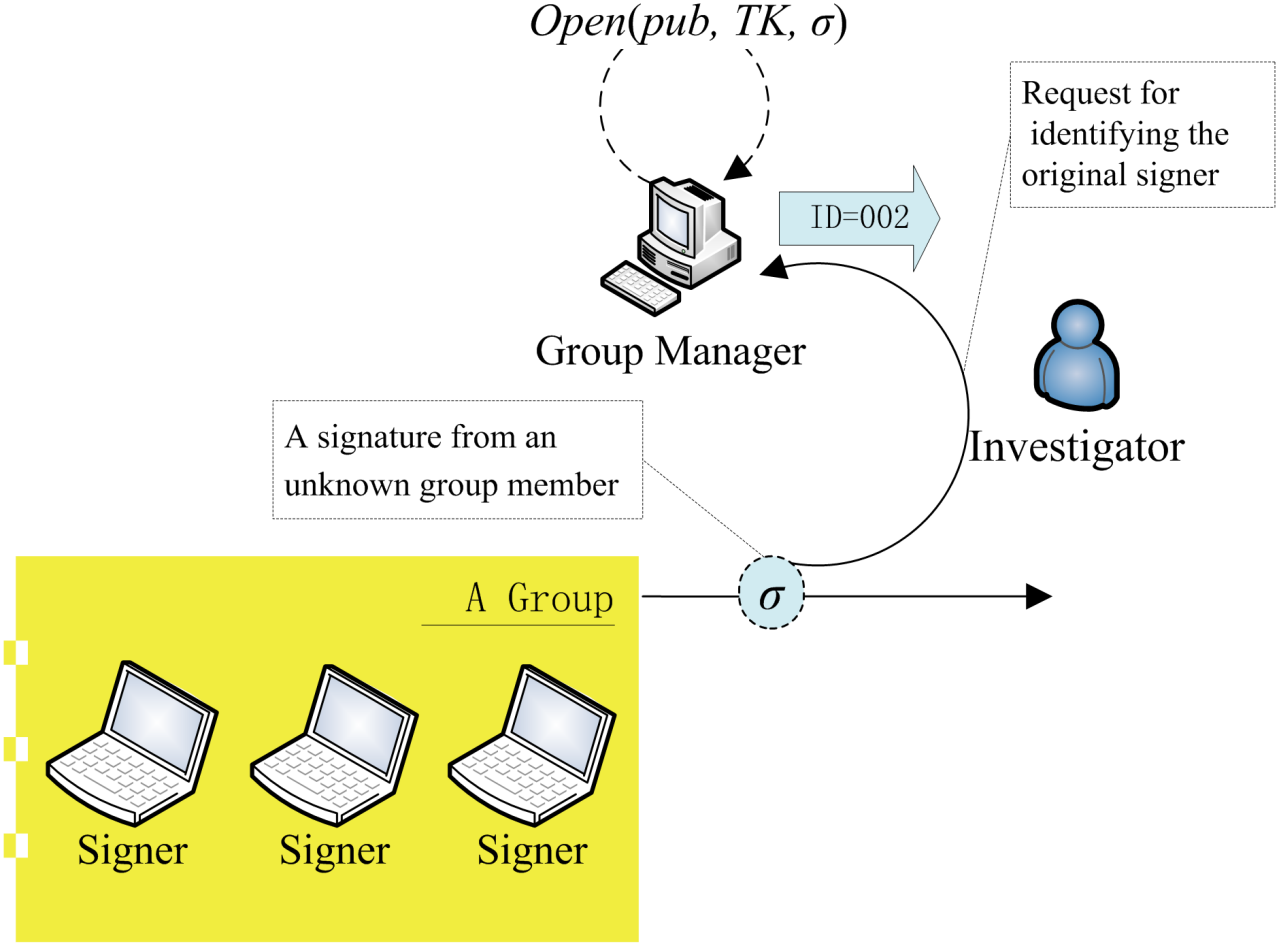
The usage of the Open algorithm.

As it is illustrated in Fig 3, to recover the identity of the signer, the group manager using the algorithm Open to test whether the signature is generated by a specific member of the group. If it is true, the algorithm outputs the identity of the member, otherwise, the output is ⊥.

The algorithm proceeds as follows.

**Algorithm** *Open*(*pub*, *σ*, *TK*)

Begin

 (*σ*_1_, *σ*_2,_ *σ*_3,_ *σ*_4_, *π*_1,_ *π*_2_) ← *σ*

$\left(n,\hat{e},G,G_{T},PP\right)\leftarrow pub$

 (*g*, *h*, *u*, *v'*, *v*_*1*,_ ..., *v*_*m*_ , Ω, *A*) ← *PP*

 For Each ID

$\text{IF}\left({\left(\sigma_{2}\right)}^{TK}={\left(g^{s_{\text{ID}}}\right)}^{TK}\right)$

*output* 1; *exit*;

*output* ⊥

End

#### 3.1.6. EKGen

The encryption key generation algorithm EKGen takes the public parameters *pub* as input, and generates a key pair (*PK*_*e*_, *SK*_*e*_) as follows. Note that for each user, the TA should provide a certification for the public encryption key *PK*_*e*_.

**Algorithm** *EKGen*(*pub*)

Begin

$\left(n,\hat{e},G,G_{T},PP\right)\leftarrow pub$

 (*g*,*h*,*u*,*v′*,*v*_1_,*…*,*v*_*m*,_Ω,*A*)←*PP*

$a,b\overset{\$}{\leftarrow }Z_{n}$

*PK*_*e*_←(*g* ^*a*^,*g* ^*b*^),*SK*_*e*_←(*a*,*b*)

*output*(*PK*_*e*,_ *SK*_*e*_)

End

#### 3.1.7. Enc and Dec

The encryption algorithm Enc takes the public parameters *pub*, the encryption key *PK*_*e*_, and a (encoded) plaintext *M* ∈ *G* as input. The algorithm encrypts the plaintext and then outputs the ciphertext as follows.

#### Remark 3

For simplicity, we use $Enc_{PK_{e}}(\cdot )$ to denote the encryption algorithm while the encryption key is *PK*_*e*_. Similarly, we use $Dec_{SK_{e}}(\cdot )$ to denote the decryption algorithm while the decryption key is *SK*_*e*_.

**Algorithm** $Enc_{PK_{e}}(M)$

Begin

$\left(n,\hat{e},G,G_{T},PP\right)\leftarrow pub$

 (*g*, *h*, *u*, *v*', *v*_1_,…, *v*_*m*_, Ω, *A*) ← *PP*

$x,y\overset{\$}{\leftarrow }Z_{n}$

 (*PK*_*e*,0_, *PK*_*e*,1_) ← *PK*_*e*_

*C*←(PK_e,0_^x^, PK_e,1_^y^, g^x+y^ ⋅ M)

*Output C*

End

The decryption algorithm Dec works as follows.

**Algorithm** *Dec_SK_e__(C)*

Begin

$\left(n,\hat{e},G,G_{T},PP\right)\leftarrow pub$

 (*C*_*1*_, *C*_*2*_, *C*_*3*_) ← *C*

 (*a*, *b*) ← *SK*_*e*_

$M\leftarrow C_{3}/\left(C_{1}{}^{\frac{1}{a}}\cdot C_{2}{}^{\frac{1}{b}}\right)$

*output M*

End

### 3.2. The Encrypted Group Signature Functionality

The encrypted group signature (EGS) functionality $EGS_{pub,sk,PK_{e}}$, with respect to a tuple *pub* that consists of system parameters and public values, the signing key *sk*, and the encryption key *PK*_*e*_, works as follows.

**Algorithm** $EGS_{pub,sk,PK_{e}}(M)$

Begin

 IF(*M = NULL*)

*output* (*pub, PK_e_*)

 Else

  (*σ*_1_, *σ*_2,_ *σ*_3,_ *σ*_4_, *π*_1,_ *π*_2_) ← *Sign*(*pub*,*sk*,*M*)

$\begin{array}{cc} output: & \left(\begin{array}{l} Enc_{PK_{e}}\left(\sigma_{1}\right),Enc_{PK_{e}}\left(\sigma_{2}\right),\\ Enc_{PK_{e}}\left(\sigma_{3}\right),Enc_{PK_{e}}\left(\sigma_{4}\right),\\ Enc_{PK_{e}}\left(\pi_{1}\right),Enc_{PK_{e}}\left(\pi_{2}\right) \end{array}\right) \end{array}$

End

**Fig 4:**
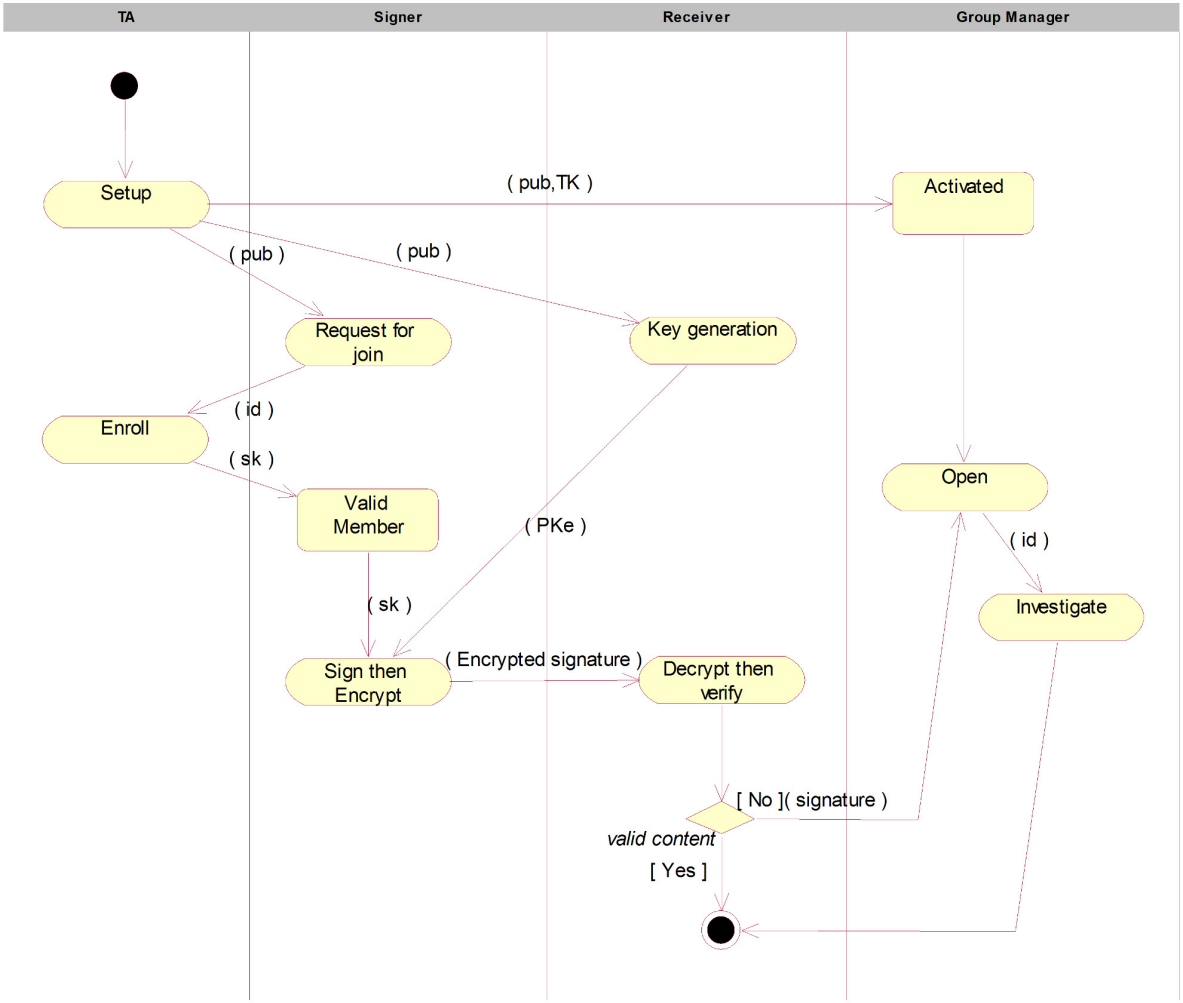
The activity diagram.

The activities of the overall scheme and the EGS functionality, include setting up the system, activating a group manager, enrolling a group member, generating and verification of a encrypted group signature, and opening the signature, are illustrated in Fig 4.

### 3.3. Efficiency Analysis

In Table 2, we list the numbers of various operations that are needed to perform each algorithm in section 3.1 and 3.2 column by column. It is shown from the table that the scheme has high efficiency in general, as the time-consuming pairing operation is only used in the Verify algorithm.

**Table 2:** Efficiency of the algorithms (listed in number of operations).

		Setup	Enroll	Sign	Verify	Open	EKGen	Enc	Dec	EGS
In *Z*_*n*_	Rand	2	1	5	0	0	2	2	0	17
	Add	0	1	0	0	0	0	1	0	6
	Mult	0	1	1	0	0	0	0	0	1
	Inv	0	1	0	0	0	0	0	2	0
	Neg	0	0	2	0	0	0	0	0	0
In *G*	Rand	0	0	0	0	0	0	0	0	0
	Mult	0	0	2m+9	m+1	0	0	1	2	2m+15
	Exp	2	3	2m+12	m	1	2	3	2	2m+30
	Inv	0	0	0	0	0	0	0	2	2
In *G*_*T*_	Mult	0	0	0	3	0	0	0	0	0
	Inv	0	0	0	3	0	0	0	0	0
*G* ^2^→*G*_*T*_	Pair	1	0	0	6	0	0	0	0	0

“Rand” denotes the operation that generates a random element of the group or ring. “Add” and “Mult” denote the addition and multiplication, respectively. “Neg” denotes the operation that generates an addictive inverse and “Inv” denotes the operation that generates a multiplicative inverse. “Pair” denotes the pairing operation.

#### Remark 4

The algorithm Setup also needs some operations that have not been listed in Table 2, such as generation of groups and large prime numbers, and random selection of group generators.

#### Remark 5

The algorithm Open can be done in constant time. Because the value ${\left(g^{s_{\text{ID}}}\right)}^{q}$ can be calculated once and for all for each user, we can construct a lookup table of ${\left(g^{s_{\text{ID}}}\right)}^{q}$ for all users in the group. With the help of the lookup table, the algorithm “Open” only needs to compute (*σ*_2_)^*q*^.

In the next section, we introduce an obfuscator for the proposed EGS functionality, and prove the correctness and security of the obfuscator.

## A Secure Obfuscator for the Special EGS Functionality

In this section, we introduce a secure obfuscator for the special EGS functionality. The proposed obfuscator can either be deployed in a physically secure device or in the signer’s host.

**Fig 5:**
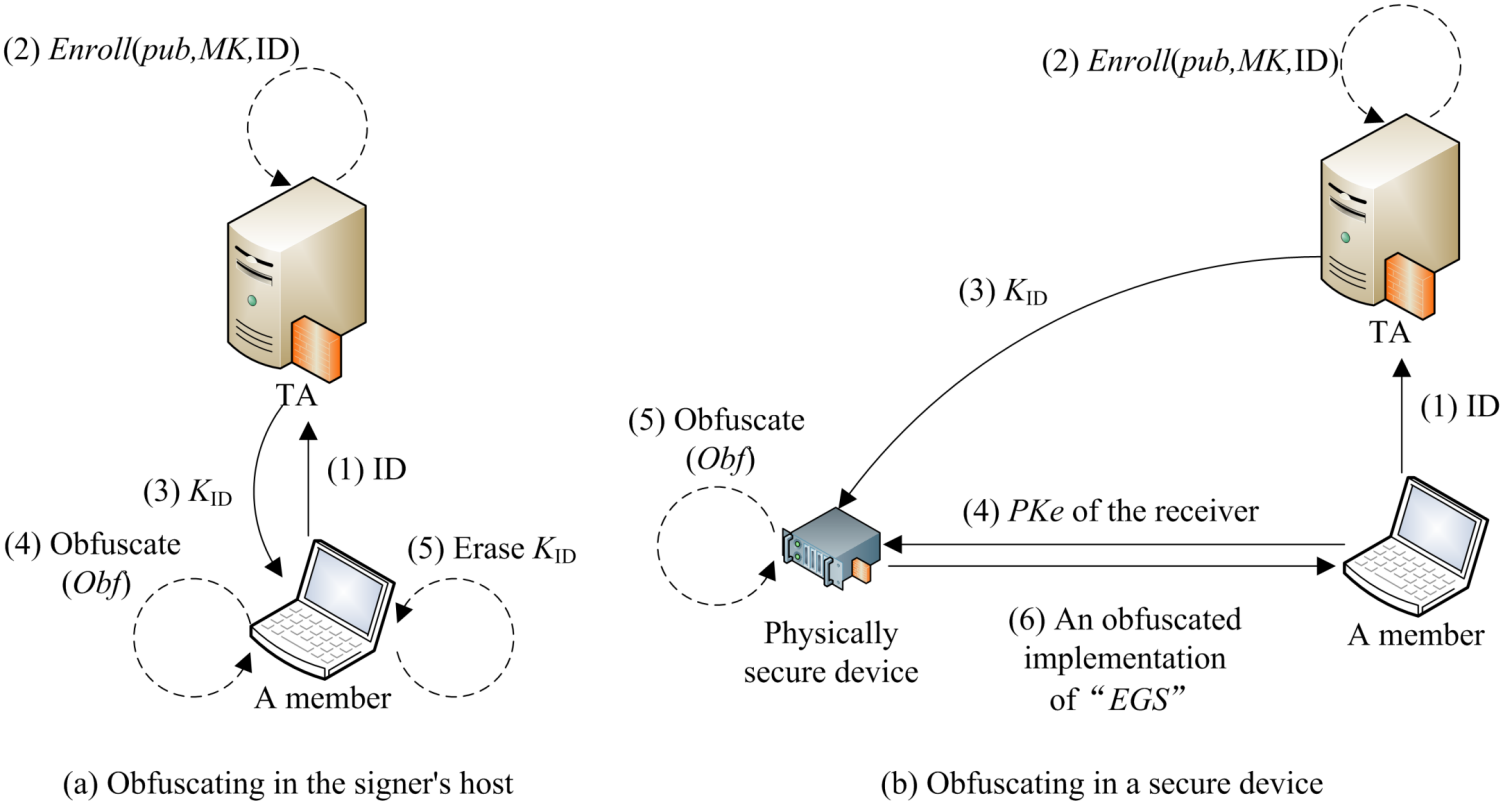
The workflow of obfuscation.

Fig 5 illustrates the workflow of obfuscation.

### 4.1. Design of the Obfuscator

In this subsection, we propose an obfuscator Obf_*EGS*_ for the $C_{pub,sk,PK_{e}}$ that implements the encrypted group signature (EGS) functionality as follows.

${\text{Obf}}_{EGS}\left(C_{pub,sk,PK_{e}}\right)$

(*PK*_*e*,*0*_,*PK*_*e*,*1*_)←*PK*_*e*_

(*K*_1_,*K*_2_,*K*_3_)←*sk*

$\left(n,\hat{e},G,G_{T},PP\right)\leftarrow pub$

$x_{1},y_{1}\overset{\$}{\leftarrow }Z_{n},x_{2},y_{2}\overset{\$}{\leftarrow }Z_{n},x_{3},y_{3}\overset{\$}{\leftarrow }Z_{n}$

$\tilde{K_{1}}\leftarrow g^{x_{1}+y_{1}}\cdot K_{\text{1}},\tilde{K_{2}}\leftarrow g^{x_{2}+y_{2}}\cdot K_{2},\;\tilde{K_{3}}\leftarrow g^{x_{3}+y_{3}}\cdot K_{3}$

$C_{x_{1}}\leftarrow PK_{e,0}{}^{x_{1}},C_{y_{1}}\leftarrow PK_{e,1}{}^{y_{1}}$

$C_{x_{2}}\leftarrow PK_{e,0}{}^{x_{2}},C_{y_{2}}\leftarrow PK_{e,1}{}^{y_{2}}$

$C_{x_{3}}\leftarrow PK_{e,0}{}^{x_{3}},C_{y_{3}}\leftarrow PK_{e,1}{}^{y_{3}}$

$z\leftarrow \left(C_{x_{1}},C_{y_{1}},C_{x_{2}},C_{y_{2}},C_{x_{3}},C_{y_{3}},\tilde{K_{1}},\tilde{K_{\text{2}}},\tilde{K_{\text{3}}}\right)$

Constructs and outputs an obfuscated circuit $R_{pub,z,PK_{e}}$ that does the following on the input message *M*:

Begin

 IF(*M*=*NULL*)

*output*(*pub*,*PK*_*e*_)

 Else

  (*μ*_1_…*μ*_*m*_)←*M*

  (*PK*_*e*,*0*_,*PK*_*e*,*1*_)←*PK*_*e*_

$\left(C_{x_{1}},C_{y_{1}},C_{x_{2}},C_{y_{2}},C_{x_{3}},C_{y_{3}},\tilde{K_{1}},\tilde{K_{\text{2}}},\tilde{K_{\text{3}}}\right)\leftarrow z$

$\left(n,\hat{e},G,G_{T},PP\right)\leftarrow pub$

$s\overset{\$}{\leftarrow }Z_{n}$

  For(*i*=1;*i*≤4;*i*++)

$t_{i}\overset{\$}{\leftarrow }Z_{n}$

  EndFor

  For(*i*=1;*i*≤6;*i*++)

$x_{i}{}^{*},y_{i}{}^{*}\overset{\$}{\leftarrow }Z_{n}$

   IF(*i*≤3)

$C_{x_{i}}{}^{*}\leftarrow {\left(PK_{e,0}\right)}^{x_{i}{}^{*}}\cdot C_{x_{i}}$

$C_{y_{i}}{}^{*}\leftarrow {\left(PK_{e,1}\right)}^{y_{i}{}^{*}}\cdot C_{y_{i}}$

   Else IF(*i*=5)

$C_{x_{i}}{}^{*}\leftarrow {\left(PK_{e,1}\right)}^{x_{i}{}^{*}}\cdot C_{x_{1}}{}^{t_{2}}\cdot C_{x_{2}}{}^{t_{1}}$

$C_{y_{i}}{}^{*}\leftarrow {\left(PK_{e,1}\right)}^{y_{i}{}^{*}}\cdot C_{y_{1}}{}^{t_{2}}\cdot C_{y_{2}}{}^{t_{1}}$

   Else

$C_{x_{i}}{}^{*}\leftarrow {\left(PK_{e,0}\right)}^{x_{i}{}^{*}}$

$C_{y_{i}}{}^{*}\leftarrow {\left(PK_{e,1}\right)}^{y_{i}{}^{*}}$

  EndFor

$\tilde{\theta_{\text{1}}}\leftarrow \tilde{K_{1}}{,}_{}\tilde{\theta_{\text{2}}}\leftarrow \tilde{K_{2}}$

$s\overset{\$}{\leftarrow }Z_{n}$

$\tilde{\theta_{\text{3}}}\leftarrow \tilde{K_{3}}\cdot {\left(v'\overset{m}{\underset{i=1}{\prod }}v_{i}^{\mu_{i}}\right)}^{s}$

$\tilde{\theta_{\text{4}}}\leftarrow g^{-s}$

  For(*i*=1;*i*≤4;*i*++)

$\tilde{\sigma_{i}}\leftarrow g^{x_{i}{}^{*}+y_{i}{}^{*}}\cdot \tilde{\theta_{i}}\cdot h^{t_{i}}$

  EndFor

$\tilde{\pi_{1}}\leftarrow g^{x_{5}{}^{*}+y_{5}{}^{*}}\cdot h^{t_{1}t_{2}}\cdot {\left(\tilde{\theta_{1}}\right)}^{t_{2}}\cdot {\left(\tilde{\theta_{2}}\Omega \right)}^{t_{1}}$

$\tilde{\pi_{2}}\leftarrow g^{x_{6}{}^{*}+y_{6}{}^{*}}\cdot u^{t_{2}}\cdot g^{-t_{3}}\cdot {\left(v'\overset{m}{\underset{i=1}{\prod }}v_{i}^{\mu_{i}}\right)}^{t_{4}}$

$\begin{array}{cc} output & {\langle C_{x_{i}}{}^{*},C_{y_{i}}{}^{*},\tilde{\sigma_{i}}\rangle }_{\left(i=1,2,3,4\right)}, \end{array}\langle C_{x_{5}}^{}{}^{*},C_{y_{5}}^{}{}^{*},\tilde{\pi_{1}}\rangle ,\langle C_{x_{6}}{}^{*},C_{y_{6}}{}^{*},\tilde{\pi_{2}}\rangle$

End

In Table 3, we list the numbers of various operations that are needed in an obfuscating transformation. Although the obfuscator looks complicated, it remains in high efficiency because the time-consuming pairing operation is never used and the complexity has nothing to do with the number of signers.

**Table 3:** Efficiency of the obfuscator.

Operation	In *Z*_*n*_					In *G*			
	Rand	Add	Mult	Inv	Neg	Rand	Mult	Exp	Inv
Number	24	9	7	0	2	0	2m+29	2m+43	0

“Rand” denotes the operation that generates a random element of the group or ring. “Add” and “Mult” denote the addition and multiplication, respectively. “Neg” denotes the operation that generates an addictive inverse and “Inv” denotes the operation that generates a multiplicative inverse.

In the next two subsections, we study the “correctness” and “security” of the obfuscator respectively.

### 4.2. Preserving Functionality

The correctness of an obfuscator Obf requires that, on a circuit *C*, Obf(*C*) behaves identically to *C* on all inputs with probability 1. Formally, this property is called “Preserving Functionality” as described in Definition 1 [34,35].

#### Definition 1. Preserving Functionality

A PPT machine Obf is a circuit obfuscator for a class of probabilistic circuits ℂ = {ℂ_*λ*_}_*λ*∈ℕ_ if, for every probabilistic circuit *C* ∈ ℂ_*λ*_, (2) holds:

Pr[C'←Obf(C) :∀x,StaDiff(C(x), C'(x))=0] = 1 (2)

where the statistical difference StaDiff between *C*(*x*) and *C*′(*x*) is given by (1).

**Theorem 1**. (Preserving Functionality). Consider any circuit $C_{pub,sk_{\text{ID}},PK_{e}}\in C_{\lambda }$ and let circuit $R_{pub,z,PK_{e}}={\text{Obf}}_{EGS}\left(C_{pub,sk,PK_{e}}\right)$. On every possible input, the output distributions of $C_{pub,sk,PK_{e}}$ and $R_{pub,z,PK_{e}}$ are identical.

**Proof**. Suppose that *PK*_*e*_ = (*g* ^*a*^, *g* ^*b*^). On an arbitrary message *M*≠*NULL*, the output of $C_{pub,sk,PK_{e}}$ is (*c*_*i*_ = *Enc*(*σ*_*i*_, *PK*_*e*_)_i = 1,2,3,4_, *c*_*i*+4_ = *Enc*(*π*_*i*_, *PK*_*e*_)_*i* = 1,2_) where (*σ*_1_, *σ*_2_, *σ*_3_, *σ*_4_, *π*_1_, *π*_2_) = *Sign*(*M*, *sk*_ID_). Let *sk*_ID_ = (*K*_1_, *K*_2_, *K*_3_) and *M* = (*μ*_1_…*μ*_*m*_) ∈ {0,1}^*m*^. We have

$$c_{1}=g^{r_{1}a},g^{s_{1}b},g^{r_{1}+s_{1}}\cdot K_{\text{1}}\cdot h^{t_{1}}$$ (3)

$$c_{2}=g^{r_{2}a},g^{s_{2}b},g^{r_{2}+s_{2}}\cdot K_{\text{2}}\cdot h^{t_{2}}$$ (4)

$$c_{3}=g^{r_{3}a},g^{s_{3}b},g^{r_{3}+s_{3}}\cdot K_{3}\cdot {\left(v'\prod_{i=1}^{m}v_{i}^{\mu_{i}}\right)}^{s}\cdot h^{t_{3}}$$ (5)

$$c_{4}=g^{r_{4}a},g^{s_{4}b},g^{r_{4}+s_{4}}\cdot g^{-s}\cdot h^{t_{4}}$$ (6)

$$c_{5}=g^{r_{5}a},g^{s_{5}b},g^{r_{5}+s_{5}}\cdot h^{t_{1}t_{2}}\cdot {\left(K_{1}\right)}^{t_{2}}\cdot {\left(K_{2}\Omega \right)}^{t_{1}}$$ (7)

$$c_{6}=g^{r_{6}a},g^{s_{6}b},g^{r_{6}+s_{6}}\cdot u^{t_{2}}\cdot g^{-t_{3}}\cdot {\left(v'\prod_{i=1}^{m}v_{i}^{\mu_{i}}\right)}^{t_{4}}$$ (8)

where *s*, *t*_1_, *t*_2_, *t*_3_, *t*_4_, *r*_1_,…, *r*_6_, *s*_1_,…, *s*_6_ are uniformly random elements of *Z*_*n*_.

Because $R_{pub,z,PK_{e}}={\text{Obf}}_{EGS}\left(C_{pub,sk,PK_{e}}\right)$, we have:

$$z=\left(C_{x_{1}},C_{y_{1}},C_{x_{2}},C_{y_{2}},C_{x_{3}},C_{y_{3}},\tilde{K_{1}},\tilde{K_{\text{2}}},\tilde{K_{\text{3}}}\right)$$ (9)

where

$$\begin{array}{l} \tilde{K_{1}}=g^{x_{1}+y_{1}}\cdot K_{\text{1}}{,}_{}\tilde{K_{2}}=g^{x_{2}+y_{2}}\cdot K_{2}{,}_{}\tilde{K_{3}}=g^{x_{3}+y_{3}}\cdot K_{3}\\ C_{x_{1}}=g^{ax_{1}}{,}_{}C_{y_{1}}=g^{by_{1}}\\ C_{x_{2}}=g^{ax_{2}}{,}_{}C_{y_{2}}=g^{by_{2}}\\ C_{x_{3}}=g^{ax_{3}}{,}_{}C_{y_{3}}=g^{by_{3}} \end{array}$$ (10)

In (10), *x*_1_, *y*_1_, *x*_2_, *y*_2_, *x*_3_, *y*_3_ are uniformly random elements of *Z*_*n*_.

For the same input *M*, suppose that the output of $R_{pub,z,PK_{e}}$ is

$$\left(c_{i}{'}_{\left(i=1,2,3,4,5,6\right)}\right)=\left({\langle C_{x_{i}}{}^{*},C_{y_{i}}{}^{*},\tilde{\sigma_{i}}\rangle }_{\left(i=1,2,3,4\right)},\langle C_{x_{5}}^{}{}^{*},C_{y_{5}}^{}{}^{*},\tilde{\pi_{1}}\rangle ,\langle C_{x_{6}}{}^{*},C_{y_{6}}{}^{*},\tilde{\pi_{2}}\rangle \right)$$ (11)

Consequently, we have:

c1'=〈Cx1*,Cy1*,σ1˜〉=〈(PKe,0)x1*⋅Cx1,(PKe,1)y1*⋅Cy1,gx1*+y1*⋅K1˜⋅ht1*〉=〈gax1*+ax1,gby1*+by1,gx1*+y1*+x1+y1⋅K1⋅ht1*〉=〈ga(x1*+x1),gb(y1*+y1),g(x1*+x1)+(y1*+y1)⋅K1⋅ht1*〉 (12)

c2'=〈Cx2*,Cy2*,σ2˜〉=〈ga(x2*+x2),gb(y2*+y2),g(x1*+x1)+(y1*+y1)⋅K1⋅ht2*〉 (13)

c3'=〈Cx3*,Cy3*,σ3˜〉=〈gax1*+ax1,gby1*+by1,gx1*+y1*+x1+y1⋅K1⋅(v'∏i=1mviμi)s*⋅ht3*〉=〈ga(x3*+x3),gb(y3*+y3),g(x3*+x3)+(y3*+y3)⋅K1⋅(v'∏i=1mviμi)s*⋅ht3*〉 (14)

c4'=〈Cx4*,Cy4*,σ4˜〉=〈gax4*,gby4*,gx4*+y4*⋅g−s*⋅ht4*〉 (15)

c5'=〈Cx5*,Cy5*,π1˜〉=〈(PKe,1)xi*⋅Cx1⋅Cx2,(PKe,1)yi*⋅Cy1⋅Cy2,gx5*+y5*⋅ht1*t2*⋅(θ1˜)t2*⋅(θ2˜Ω)t1*〉=〈gax5*⋅gax1t2*⋅gax2t1*,gay5*⋅gay1t2*⋅gay2t1*,gx5*+y5*⋅ht1*t2*⋅(K1˜)t2*⋅(K2˜Ω)t1*〉=〈ga(x5*+x1t2*+x2t1*),gb(y5*+y1t2*+y2t1*),gx5*+y5*⋅ht1*t2*⋅(gx1+y1⋅K1)t2*⋅(gx2+y2⋅K2Ω)t1*〉=〈ga(x5*+x1t2*+x2t1*),gb(y5*+y1t2*+y2t1*),gx5*+y5*⋅gx1t2*+y1t2*⋅gx2t1*+y2t1*⋅ht1*t2*⋅(K1)t2*⋅(K2Ω)t1*〉=〈ga(x5*+x1t2*+x2t1*),gb(y5*+y1t2*+y2t1*),g(x5*+x1t2*+x2t1*)+(y5*+y1t2*+y2t1*)⋅ht1*t2*⋅(K1)t2*⋅(K2Ω)t1*〉 (16)

c6'=〈Cx6*,Cy6*,π2˜〉=〈gax6*,gby6*,gx6*+y6*⋅ut2*⋅g−t3*⋅(v'∏i=1mviμi)t4*〉 (17)

In the above six equations, $s^{*},t_{1}{}^{*},t_{2}{}^{*},t_{3}{}^{*},t_{4}{}^{*},x_{1}{}^{*},\cdots ,x_{6}{}^{*},y_{1}{}^{*},\cdots ,y_{6}{}^{*}$ are uniformly random elements of *Z*_*n*_.

Let $V=v'\prod_{i=1}^{m}v_{i}^{\mu_{i}}$, the two tuples of ciphertexts are listed in Table 4.

**Table 4:** Comparison of ciphertexts.

i	c_i_	ci'
1	$g^{r_{1}a},g^{s_{1}b},g^{r_{1}+s_{1}}\cdot K_{\text{1}}\cdot h^{t_{1}}$	$g^{a\left(x_{1}{}^{*}+x_{1}\right)},g^{b\left(y_{1}{}^{*}+y_{1}\right)},g^{\left(x_{1}{}^{*}+x_{1}\right)+\left(y_{1}{}^{*}+y_{1}\right)}\cdot K_{1}\cdot h^{t_{1}{}^{*}}$
2	$g^{r_{2}a},g^{s_{2}b},g^{r_{2}+s_{2}}\cdot K_{\text{2}}\cdot h^{t_{2}}$	$g^{a\left(x_{2}{}^{*}+x_{2}\right)},g^{b\left(y_{2}{}^{*}+y_{2}\right)},g^{\left(x_{1}{}^{*}+x_{1}\right)+\left(y_{1}{}^{*}+y_{1}\right)}\cdot K_{1}\cdot h^{t_{2}{}^{*}}$
3	$g^{r_{3}a},g^{s_{3}b},g^{r_{3}+s_{3}}\cdot K_{3}\cdot V^{s}\cdot h^{t_{3}}$	$g^{a\left(x_{3}{}^{*}+x_{3}\right)},g^{b\left(y_{3}{}^{*}+y_{3}\right)},g^{\left(x_{3}{}^{*}+x_{3}\right)+\left(y_{3}{}^{*}+y_{3}\right)}\cdot K_{1}\cdot V^{s^{*}}\cdot h^{t_{3}{}^{*}}$
4	$g^{r_{4}a},g^{s_{4}b},g^{r_{4}+s_{4}}\cdot g^{-s}\cdot h^{t_{4}}$	$g^{ax_{4}{}^{*}},g^{by_{4}{}^{*}},g^{x_{4}{}^{*}+y_{4}{}^{*}}\cdot g^{-s^{*}}\cdot h^{t_{4}{}^{*}}$
5	$g^{r_{5}a},g^{s_{5}b},g^{r_{5}+s_{5}}\cdot h^{t_{1}t_{2}}\cdot {\left(K_{1}\right)}^{t_{2}}\cdot {\left(K_{2}\Omega \right)}^{t_{1}}$	$\begin{array}{l} g^{a\left(x_{5}{}^{*}+x_{1}t_{2}{}^{*}+x_{2}t_{1}{}^{*}\right)},g^{b\left(y_{5}{}^{*}+y_{1}t_{2}{}^{*}+y_{2}t_{1}{}^{*}\right)},\\ g^{\left(x_{5}{}^{*}+x_{1}t_{2}{}^{*}+x_{2}t_{1}{}^{*}\right)+\left(y_{5}{}^{*}+y_{1}t_{2}{}^{*}+y_{2}t_{1}{}^{*}\right)}\cdot h^{t_{1}{}^{*}t_{2}{}^{*}}\cdot {\left(K_{\text{1}}\right)}^{t_{2}{}^{*}}\cdot {\left(K_{2}\Omega \right)}^{t_{1}{}^{*}} \end{array}$
6	$g^{r_{6}a},g^{s_{6}b},g^{r_{6}+s_{6}}\cdot u^{t_{2}}\cdot g^{-t_{3}}\cdot V^{t_{4}}$	$g^{ax_{6}{}^{*}},g^{by_{6}{}^{*}},g^{x_{6}{}^{*}+y_{6}{}^{*}}\cdot u^{t_{2}{}^{*}}\cdot g^{-t_{3}{}^{*}}\cdot V^{t_{4}{}^{*}}$

Clearly, the two tuples of ciphertexts are identically distributed.

If the input is a null message *M* = *NULL*, both the output of $C_{pub,sk,PK_{e}}$ and the output of $R_{pub,z,PK_{e}}$ are (*pub*, *PK*_*e*_).

This ends the proof.

### 4.3. Security Properties

#### 4.3.1. Security notions for the EGS functionality and the obfuscator

Average-Case Secure Virtual Black-box Property (ACVBP) was proposed in [35], and it was extended to ACVBP w.r.t. Dependent Oracles in [34]. The generalization allows distinguishers to have sampling access not only to <<*C*>> but also to a set of oracles dependent on *C*.

#### Definition 2

A circuit obfuscator Obf for ℂ satisfies the ACVBP w.r.t. dependent oracle set *T* if the following condition holds: There exists a PPT oracle machine *S* (simulator) such that, for every PPT oracle machine *𝓓* (distinguisher), every polynomial *f*, all sufficiently large *λ* ∈ ℕ, and every z ∈ {0,1}^poly(*λ*)^,

$$\left|\text{Pr}\left[\begin{array}{cc} \begin{array}{l} C\leftarrow {\mathbb{C}}_{\lambda };\\ C'\leftarrow \text{Obf}\left(C\right);\\ b\leftarrow {𝓓}^{<<C,T\left(C\right)>>}\left(C',z\right) \end{array} & :b=1 \end{array}\right]-\text{Pr}\left[\begin{array}{cc} \begin{array}{l} C\leftarrow {\mathbb{C}}_{\lambda };\\ C''\leftarrow S^{<<C>>}\left(1^{\lambda },z\right);\\ b\leftarrow {𝓓}^{<<C,T\left(C\right)>>}\left(C'',z\right) \end{array} & :b=1 \end{array}\right]\right|<\frac{1}{f\left(\lambda \right)}$$ (18)

where *𝓓* ^<<*C*, *T*(*C*)>>^ means that *𝓓* has sampling access to all oracles contained in *T*(*C*) in addition to *C*.

To the best of our knowledge, in obfuscating sig-then-encrypt functionalities, *T*(*C*) is always assigned to the signature function, such as in [34,36,38,41,49,50]. However, we have to investigate the effects of collision attacks from some members of the same group against the proposed obfuscator. In this scenario, the adversary against the obfuscator can get the signing key of a corrupted group member, that is, the adversary can query the *Enroll* oracle on the identity of a corrupted member. Because there are some restrictions on these kinds of queries, we define a set of restricted oracles dependent on *C* as *𝓡*(*C*). Each element of *𝓡*(*C*) is an oracle-restrictions pair. For example, in this paper *𝓡*(*C*) = {(*Enroll*, *id*≠ID)}. Conventionally, when *𝓡*(*C*) consists of only one element, we omit the braces. Moreover, we suggest that the restrictions could be written as superscripts of the oracle, e.g., *Enroll* ^[*id*≠ID]^.

Based on the above intuition, we extended ACVBP w.r.t. Dependent Oracles (**Definition 2**) to ACVBP w.r.t. Dependent Oracles and Restricted Dependent Oracles as follows.

#### Definition 3

A circuit obfuscator Obf for ℂ satisfies the ACVBP w.r.t. dependent oracle set *T* and restricted dependent oracle set *𝓡* if the following condition holds: There exists a PPT oracle machine *S* (simulator) such that, for every PPT oracle machine *𝓓* (distinguisher), every polynomial *f*, all sufficiently large *λ* ∈ ℕ, and every z ∈ {0,1}^poly(*λ*)^,

$$\left|\begin{array}{l} \text{Pr}\left[\begin{array}{cc} \begin{array}{l} C\leftarrow {\mathbb{C}}_{\lambda };\\ C'\leftarrow \text{Obf}\left(C\right);\\ b\leftarrow {𝓓}^{<<C,T\left(C\right),𝓡\left(C\right)>>}\left(C',z\right) \end{array} & :b=1 \end{array}\right]\\ -\text{Pr}\left[\begin{array}{cc} \begin{array}{l} C\leftarrow {\mathbb{C}}_{\lambda };\\ C''\leftarrow S^{<<C>>}\left(1^{\lambda },z\right);\\ b\leftarrow {𝓓}^{<<C,T\left(C\right),𝓡\left(C\right)>>}\left(C'',z\right) \end{array} & :b=1 \end{array}\right] \end{array}\right|<\frac{1}{f\left(\lambda \right)}$$ (19)

where *𝓓* ^<<*C*, *T*(*C*), *𝓡*(*C*)>>^ means that *𝓓* has sampling access to all oracles contained in *T*(*C*) and *𝓡*(*C*) in addition to *C*.

**Fig 6:**
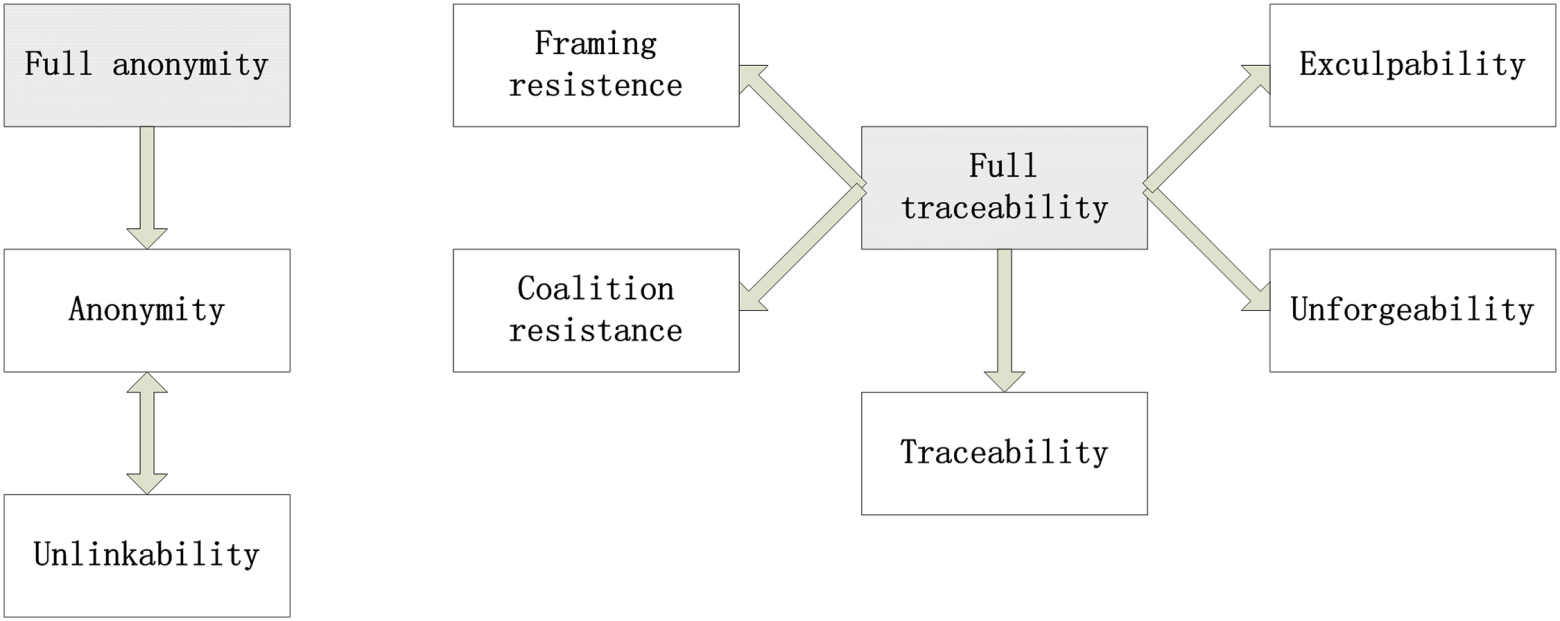
Security notions of group signature schemes.

Besides the security notion for the obfuscator in WBAC, we should also provide security notions for the EGS functionality. There is a number of existing security notions of group signature schemes. Fortunately, as shown in Fig 6, there are two cores that imply other security notions as it was discussed in [2]. Hence, we focus on the full traceability (FT) and full anonymity (FA). Note that we use the CPA-full-anonymity[46] instead of the CCA2-full-anonymity[2].

We formally define full-traceability (FT w.r.t. EGS Functionality) using the following experiment.

$Exp_{EGS,𝓕}^{Trace}(\lambda ,k,m)$

Begin

$\left(pub,priv\right)=\left(\left(n,\hat{e},G,G_{T},PP\right),\left(MK,TK\right)\right)\leftarrow Setup\left(1^{\lambda },1^{k},1^{m}\right)$

 (*PK*_*e*_, *SK*_*e*_) ← *EKGen*(*pub*)

*st←*(*pub*, *TK*, *PK*_*e*_);Θ←∅;*L* ← ∅;Γ←ε;*count*←*true*

While(*cont* = *true*) do

$\left(cont,st,id)\overset{\$}{\leftarrow }{𝓕}^{\langle \langle Sign_{sk_{\text{ID}}}\left(\right)\rangle \rangle }(choose,st,\Gamma \right)$

$L\leftarrow L\cup \left\{\left(M,\text{ID}\right)|Sign_{sk_{\text{ID}}}(M)\text{ has been queried by }𝓕\right\}$

  IF(*cont* = *true*)

   Θ←Θ∪{*id*};Γ ← *Enroll*(*pub*, *MK*, *id*)

 EndWhile

$\left(M,\sigma )\overset{\$}{\leftarrow }{𝓕}^{\langle \langle Sign_{sk_{\text{ID}}}\left(\right)\rangle \rangle }(guess,st\right)$

 IF(0 = *Verify*(*pub*, σ, *M*)) return 0;

 IF(⊥ = *Open*(*pub*, *TK*, σ, *M*)) return 0;

 IF(∃*id* ∈ [0,…, 2^*k*^ – 1], s.t. *id* = *Open*(*TK*, σ, *M*) ∧ *id* ∉ Θ ∧ (*M*, ID) ∉ *L*)

  return 0

 Else

  return 1

End

#### Definition 4

(FT w.r.t. EGS Functionality). Let (*Setup*, *Enroll*, *Sign*, *Verify*) and (*EKGen*, *Enc*, *Dec*) be a pair of group signature and public key encryption schemes. The group signature scheme is FT w.r.t. the EGS functionality if the following condition holds: For every PPT oracle machine *𝓕* (adversary), every polynomial *f*, all sufficiently large *λ* ∈ ℕ, and every *st* ∈ {0,1}^poly(*λ*)^,

$$\text{Pr}\left[Exp_{EGS,𝓕}^{Trace}\left(\lambda ,k,m\right)\right]<\frac{1}{f\left(\lambda \right)}$$ (20)

We formally define full-anonymity (FA w.r.t. EGS Functionality) using the following experiment.

$Exp_{EGS,𝓕}^{CPA-Anon-b}(\lambda ,k,m)$

Begin

$\left(pub,priv\right)=\left(\left(n,\hat{e},G,G_{T},PP\right),\left(MK,TK\right)\right)\leftarrow Setup\left(1^{\lambda },1^{k},1^{m}\right)$

 (*PK*_*e*_,*SK*_*e*_) ← *EKGen*(*pub*)

*SK* ← ∅;*id* ← 0

 While(*id* < 2^*k*^) do

*SK* ← *SK* ∪ *Enroll*(*pub*,*MK*,*id*);*id* ← *id* + 1

 EndWhile

$\left(st,id_{0},id_{1},M\right)\overset{\$}{\leftarrow }𝓕(choose,pub,SK,PK_{e})$

$\sigma \leftarrow Sign_{sk_{id_{b}}}(M)$

$d\overset{\$}{\leftarrow }{𝓕}^{\langle \langle Sign_{sk}\left(\right)\rangle \rangle }(guess,st,\sigma )$

 return *d*

End

#### Definition 5

(FA w.r.t. EGS Functionality). Let (*Setup*, *Enroll*, *Sign*, *Verify*) and (*EKGen*, *Enc*, *Dec*) be a pair of group signature and public key encryption schemes. The group signature scheme is full anonymous w.r.t. the EGS functionality if the following condition holds: For every PPT oracle machine *𝓕* (adversary), every polynomial *f*, all sufficiently large *λ* ∈ ℕ, and every *st* ∈ {0,1}^poly(*λ*)^,

$$\left|\text{Pr}\left[d\leftarrow Exp_{EGS,𝓕}^{CPA-Anon-b}:b=d\right]-\frac{1}{2}\right|<\frac{1}{f\left(\lambda \right)}$$ (21)

Next, we consider a pair of stronger security notions, which require that the group signature scheme is still secure even when the adversary is given an obfuscated circuit. The following experiments are used to describe the strengthened definitions of full-traceability and full-anonymity respectively.

$Exp_{EGS,{\text{Obf}}_{EGS},𝓕}^{Trace}(\lambda ,k,m)$

Begin

$\left(pub,priv\right)=\left(\left(n,\hat{e},G,G_{T},PP\right),\left(MK,TK\right)\right)\leftarrow Setup\left(1^{\lambda },1^{k},1^{m}\right)$

 (*PK_e_, SK_e_*) ← *EKGen*(*pub*)

*st* ← (*pub*, *TK*, *PK*_*e*_);*SK* ← ∅;Θ ← ∅;*L* ← ∅;*Γ* ← *ε*;*cont* ← *true*

 While(*id* < 2^*k*^) do

*SK* ← *SK* ∪ *Enroll*(*pub*, *MK*, *id*);*id* ← *id* + 1

 EndWhile

 While(*cont* = *true*) do

$\left(cont,st,id)\overset{\$}{\leftarrow }{𝓕}^{\langle \langle Sign_{sk_{\text{ID}}}\left(\right),O_{EGS}\left(\right)\rangle \rangle }(choose,st,\Gamma \right)$

$L\leftarrow L\cup \left\{\left(M,\text{ID}\right)|Sign_{sk_{\text{ID}}}(M)\text{ has been queried by }𝓕\right\}$

  IF(*cont* = *true*)

   Θ ← Θ ∪ {*id*};*Γ* ← *Enroll*(*pub*, *MK*, *id*)

 EndWhile

$\left(M,\sigma )\overset{\$}{\leftarrow }{𝓕}^{\langle \langle Sign_{sk_{\text{ID}}}\left(\right),O_{EGS}\left(\right)\rangle \rangle }(guess,st\right)$

 IF(0 = *Verify*(*pub*, *σ*, *M*)) return 0;

 IF(⊥ = *Open*(*pub*, *TK*, *σ*, *M*)) return 0;

 IF(∃*id* ∈ [0, …, 2^*k*^ - 1], s.t. *id* = *Open*(*TK*, *σ*, *M*) ∧ *id* ∉ Θ ∧ (*M*, ID) ∉ *L*)

  return 0

 Else

  return 1

End

Algorithm *O*_*EGS*_(*id*)

Begin

 Extract *sk*_*id*_ from *SK*

${\text{return Obf}}_{EGS}\left(C_{pub,sk_{id},PK_{e}}\right)$

End

$Exp_{EGS,{\text{Obf}}_{EGS},𝓕}^{CPA-Anon-b}(\lambda ,k,m)$

Begin

$\left(pub,priv\right)=\left(\left(n,\hat{e},G,G_{T},PP\right),\left(MK,TK\right)\right)\leftarrow Setup\left(1^{\lambda },1^{k},1^{m}\right)$

 (*PK*_*e*_, *SK*_*e*_) ← *EKGen*(*pub*)

*SK* ← ∅;*id* ← 0

 While(*id* < 2^*k*^) do

*SK* ← *SK* ∪ *Enroll*(*pub*, *MK*, *id*);*id* ← *id* + 1

 EndWhile

$\left(st,id_{0},id_{1},M\right)\overset{\$}{\leftarrow }𝓕(choose,pub,SK,PK_{e})$

$\sigma \leftarrow Sign_{sk_{id_{b}}}(M)$

$d\overset{\$}{\leftarrow }{𝓕}^{\langle \langle Sign_{sk}\left(\right),O_{EGS}\left(\right)\rangle \rangle }(guess,st,\sigma )$

 return *d*

End

Algorithm *O*_*EGS*_(*id*)

Begin

 Extract *sk*_*id*_ from *SK*

${\text{return Obf}}_{EGS}\left(C_{pub,sk_{id},PK_{e}}\right)$

End

Now we give definitions of full-traceability (FT) and full-anonymity (FA) w.r.t. EGS Obfuscator as follows.

#### Definition 6

(FT w.r.t. EGS Obfuscator). Let (*Setup*, *Enroll*, *Sign*, *Verify*) and (*EKGen*, *Enc*, *Dec*) be a pair of group signature and public key encryption schemes. The group signature scheme is FT w.r.t. Obf_*EGS*_ if the following condition holds: For every PPT oracle machine *𝓕* (adversary), every polynomial *f*, all sufficiently large *λ* ∈ ℕ, and every *st* ∈ {0,1}^poly(*λ*)^,

$$\text{Pr}\left[Exp_{EGS,{\text{Obf}}_{EGS},𝓕}^{Trace}\left(\lambda ,k,m\right)\right]<\frac{1}{f\left(\lambda \right)}$$ (22)

#### Definition 7

(FA w.r.t. EGS Obfuscator). Let (*Setup*, *Enroll*, *Sign*, *Verify*) and (*EKGen*, *Enc*, *Dec*) be a pair of group signature and public key encryption schemes. The group signature scheme is FA w.r.t. Obf_*EGS*_ if the following condition holds: For every PPT oracle machine *𝓕* (adversary), every polynomial *f*, all sufficiently large *λ* ∈ ℕ, and every *st* ∈ {0,1}^poly(*λ*)^,

$$\left|\text{Pr}\left[d\leftarrow Exp_{EGS,{\text{Obf}}_{EGS},𝓕}^{CPA-Anon-b}:b=d\right]-\frac{1}{2}\right|<\frac{1}{f\left(\lambda \right)}$$ (23)

#### Definition 8

A circuit obfuscator Obf for ℂ is rerandomizable (RR) w.r.t. dependent oracle set *T* and restricted dependent oracle set *𝓡* if the following condition holds: There exists a PPT oracle machine *RR* such that, for every PPT oracle machine *𝓓* (distinguisher), every polynomial *f*, all sufficiently large *λ* ∈ ℕ, and every*z* ∈ {0,1}^poly(*λ*)^, when *C* ∈ ℂ_*λ*_ and *C*′←Obf(*C*), (24) holds.

$$\left|\begin{array}{l} \text{Pr}\left[\begin{array}{cc} b\leftarrow {𝓓}^{<<C,T\left(C\right),𝓡\left(C\right)>>}\left(C',z\right) & :b=1 \end{array}\right]\\ -\text{Pr}\left[\begin{array}{cc} \begin{array}{l} C''\leftarrow RR\left(C'\right);\\ b\leftarrow {𝓓}^{<<C,T\left(C\right),𝓡\left(C\right)>>}\left(C'',z\right) \end{array} & :b=1 \end{array}\right] \end{array}\right|<\frac{1}{f\left(\lambda \right)}$$ (24)

In (24), *𝓓* ^<<*C*, *T*(*C*), *𝓡*(*C*)>>^ means that *𝓓* has sampling access to all oracles contained in *T*(*C*) and *𝓡*(*C*) in addition to *C*.

Note that if a circuit obfuscator Obf for ℂ is rerandomizable w.r.t. dependent oracle set *T* and restricted dependent oracle set *𝓡* and Obf also satisfies the ACVBP w.r.t. dependent oracle set *T* and restricted dependent oracle set *𝓡*, we say that Obf is RR&ACVBP w.r.t. dependent oracle set *T* and restricted dependent oracle set *𝓡*.

**Fig 7:**
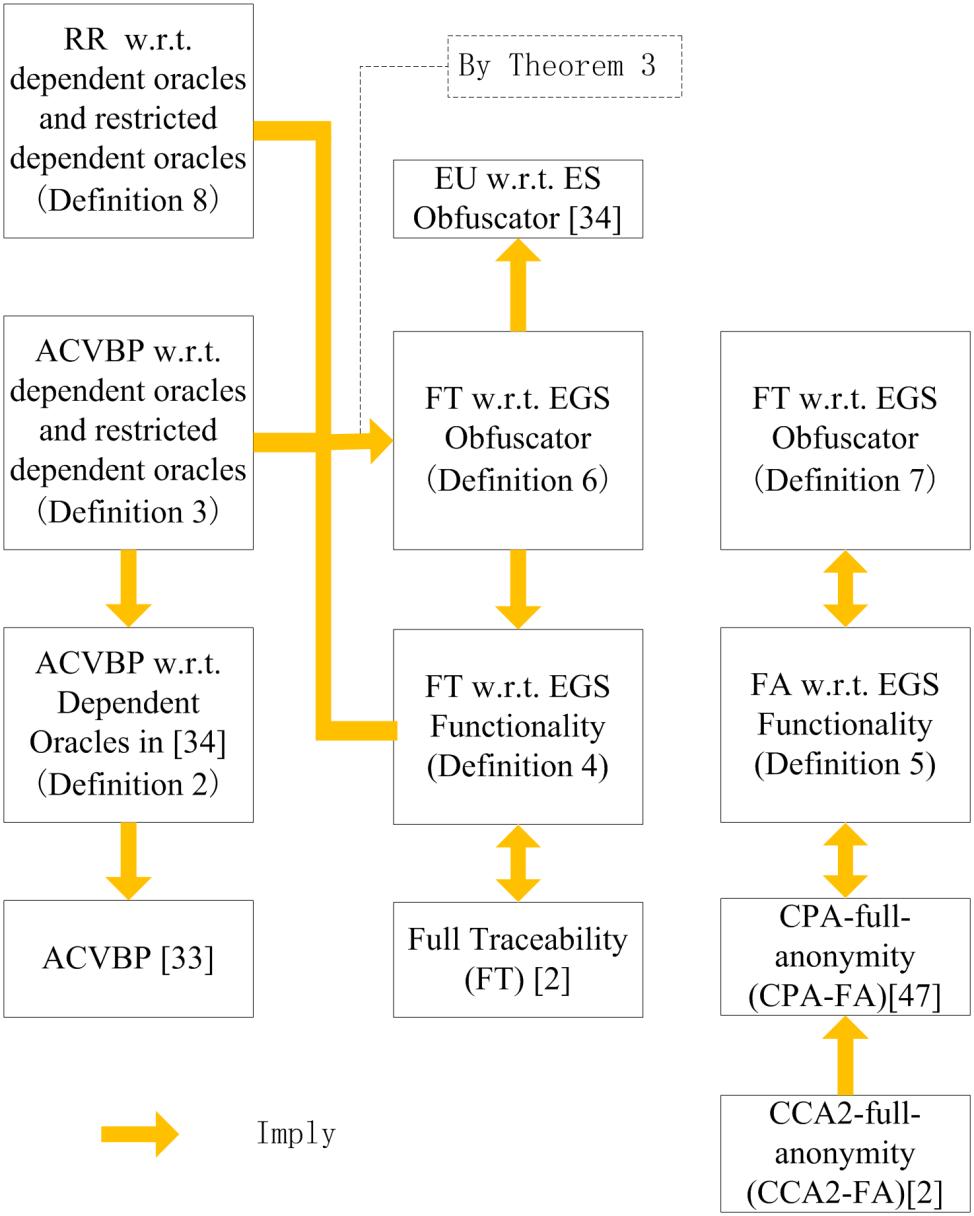
Relationships among the proposed security notions and known security notions.

There are six new security notions that are proposed in this section. Relationships among the proposed security notions and known security notions are shown in Fig 7. In Fig 7, the arrows denote the “imply” relationships. Most of the “imply” relationships are easy to verify so we omit the detail analysis, except the complex one that is investigated in Theorem 3.

As illustrated in Fig 7, some new security notions are equal to old ones. Especially, Definition 5 (FA w.r.t. EGS Obfuscator), Definition 7 (FA w.r.t. EGS functionality) and the CAP-full-anonymity (in [46]) are equivalent. Hence, from the practice point of view, it seems that Definition 5 and Definition 7 are somewhat useless because in fact depict the same security of CAP-full-anonymity. However, to provide these security notions and investigate them are necessary, both to acquire the result and to fulfill the completeness of theory.

#### 4.3.2. The main security theorem

**Theorem 2**. The proposed obfuscator Obf_*EGS*_ for the proposed EGS functionality satisfies ACVBP w.r.t. dependent oracle $T\left(C\right)=Sign_{sk_{\text{ID}}}$ and restricted dependent oracle $𝓡(C)=Enroll_{MK}^{\left[id\neq \text{ID}\right]}$.

**Proof**. We have $C=C_{pub,sk_{\text{ID}},PK_{e}}$, $T\left(C\right)=Sign_{sk_{\text{ID}}}$ and $𝓡(C)=Enroll_{MK}^{\left[id\neq \text{ID}\right]}$. Then we define a pair of probabilities in (25) and (26). They are the probabilities that *𝓓* ^<<*C*, *T*(*C*), *𝓡*(*C*)>>^ outputs 1, given the real and simulated distributions, respectively. *sk*_ID_ = (*K*_1_, *K*_2_, *K*_3_) is encrypted in the real distribution while (*K*_1_′, *K*_2_′, *K*_3_′) ∈ _$_ *G* ^3^ is encrypted in the simulated distribution. It is the only difference.

Let

$$\begin{array}{l} {\text{Pr}}_{Nick}\\ =\text{Pr}\left[\begin{array}{cc} \begin{array}{l} \left(pub,priv\right)=\left(\left(n,\hat{e},G,G_{T},PP\right),\left(MK,TK\right)\right)\leftarrow Setup\left(1^{\lambda },1^{k},1^{m}\right)\\ \left(PK_{e},SK_{e}\right)\overset{\$}{\leftarrow }EKGen\left(pub\right)\\ sk_{\text{ID}}=\left(K_{1},K_{2},K_{3}\right)\overset{\$}{\leftarrow }Enroll\left(pub,MK,\text{ID}\right)\\ C'\leftarrow {\text{Obf}}_{EGS}\left(C_{pub,sk_{\text{ID}},PK_{e}}\right)\\ b\leftarrow {𝓓}^{<<C_{pub,sk_{\text{ID}},PK_{e}}\left(\right),Sign_{sk_{\text{ID}}}\left(\right),Enroll_{MK}^{\left[id\neq \text{ID}\right]}\left(\right)>>}\left(C'\right) \end{array} & :b=1 \end{array}\right] \end{array}$$ (25)

and

$$\begin{array}{l} {\text{Pr}}_{Junk}\\ =\text{Pr}\left[\begin{array}{cc} \begin{array}{l} \left(pub,priv\right)=\left(\left(n,\hat{e},G,G_{T},PP\right),\left(MK,TK\right)\right)\leftarrow Setup\left(1^{\lambda },1^{k},1^{m}\right)\\ \left(PK_{e},SK_{e}\right)\overset{\$}{\leftarrow }EKGen\left(pub\right)\\ sk_{\text{ID}}=\left(K_{1},K_{2},K_{3}\right)\overset{\$}{\leftarrow }Enroll\left(pub,MK,\text{ID}\right)\\ C''\leftarrow Sim^{<<C_{pub,sk_{\text{ID}},PK_{e}}\left(\right)>>}\left(\right)\\ b\leftarrow {𝓓}^{<<C_{pub,sk_{\text{ID}},PK_{e}}\left(\right),Sign_{sk_{\text{ID}}}\left(\right),Enroll_{MK}^{\left[id\neq \text{ID}\right]}\left(\right)>>}\left(C''\right) \end{array} & :b=1 \end{array}\right] \end{array}$$ (26)

We construct a simulator *Sim* that works as follows

$Sim^{\langle \langle C_{pub,sk_{\text{ID}},PK_{e}}\left(\right)\rangle \rangle }\left(\;\right)$

$\left(pub,PK_{e}\right)\leftarrow C_{pub,sk_{\text{ID}},PK_{e}}\left(\;\right)$

$\left(n,\hat{e},G,G_{T},PP\right)\leftarrow pub$

(*g* ^*a*^, *g* ^*b*^) ← *PK*_*e*_

$x_{1},y_{1}\overset{\$}{\leftarrow }Z_{n},\;x_{2},y_{2}\overset{\$}{\leftarrow }Z_{n},\;x_{3},y_{3}\overset{\$}{\leftarrow }Z_{n}$

K1',K2',K3'←$G

K1˜'←gx1+y1⋅K1', K2˜'←gx2+y2⋅K2', K3˜'←gx3+y3⋅K3'

Cx1'←gax1,Cy1'←gby1

Cx2'←gax2,Cy2'←gby2

Cx3'←gax3,Cy3'←gby3

Junk←(Cx1',Cy1',Cx2',Cy2',Cx3',Cy3',K1˜',K2˜',K3˜')

$\begin{array}{cc} output & R_{pub,Junk,PK_{e}}()\text{ that works the same as }R_{pub,z,PK_{e}}() \end{array}$

For contradiction, assume that the probability that a distinguisher ${𝓓}^{<<C_{pub,sk_{\text{ID}},PK_{e}}\left(\right),Sign_{sk_{\text{ID}}}\left(\right),Enroll_{MK}^{\left[id\neq \text{ID}\right]}\left(\right)>>}$ can distinguish between *C*' and *C*" is not negligible. That is, the difference between the following two probabilities is not negligible. Without loss of generosity, we suppose that Pr_*Nick*_-Pr_*Junk*_ = *δ* > 0.

Then an adversary pair (*𝓐, 𝓑*) which breaks the indistinguishability of the linear encryption scheme is constructed as follows. *𝓐* produces a plaintext pair 〈*sk*_ID_, *sk*′〉, and the associated public setting and some global parameters of the asymmetric encryption scheme using *𝓐*.*Init* and plays the following security game with *𝓓* and *𝓑*.

Game1𝓓𝓐𝓑(st,challenge)=Init(1λ)→challengect=𝓑.CipherTextGen(challenge)↓st←ct,PKe𝓓←Rpub,ct,PKe→O<<Cpub,skID,PKe()>>O<<SignskID()>>O<<EnrollMK[id≠ID]()>>→return0 or 1Guess(ct,st)Begin⋮⋮⋮Endoutput d'

We list the usage of the algorithms that are used in Game1, i.e., *𝓐*.*Init*, *𝓐*.*OC*, *𝓐*.*OS*, *𝓐*.*OE*, *𝓐*.*Guess*, and *𝓑*.*CipherTextGen*, in Table 5.

**Table 5:** The algorithms in Game1.

Algorithm	Usage
*𝓐*.*Init*	Initiate the parameter.
*𝓐*.*OC*	Reply the $O^{<<C_{pub,sk_{\text{ID}},PK_{e}}\left(\right)>>}$ queries from *𝓓*.
*𝓐*.*OS*	Reply the $O^{<<Sign_{sk_{\text{ID}}}\left(\right)>>}$ queries from *𝓓*.
*𝓐*.*OE*	Reply the $O^{<<Enroll_{MK}^{\left[id\neq \text{ID}\right]}\left(\right)>>}$ queries from *𝓓*.
*𝓐*.*Guess*	Guess the value of *d* in *𝓑*.*CipherTextGen*
*𝓑*.*CipherTextGen*	Generate the challenge ciphertext.

The descriptions of the algorithms are as follows.

**Algorithm** *𝓐*.*Init*(1^λ^)

Begin

$\left(pub,priv\right)=\left(\left(n,\hat{e},G,G_{T},PP\right),\left(MK,TK\right)\right)\leftarrow Setup\left(1^{\lambda },1^{k},1^{m}\right)$

$sk_{\text{ID}}=\left(K_{1},K_{2},K_{3}\right)\overset{\$}{\leftarrow }Enroll\left(pub,MK,\text{ID}\right)$

sk'=(K1',K2',K3')←$G

$challenge\leftarrow \left(sk_{\text{ID}},sk',n,\hat{e},G,G_{T},g\right)$

*st* ← (*pub*,*priv*)

*output* (*st*, *challenge*)

End

#### Remark 6

We suppose that *𝓐* generates the system parameters honestly, that is, *𝓐* does not set any “backdoor” in the parameters. Otherwise, the system parameters could be generated by a trusted third party.

#### Remark 7

After *𝓐* is initialized, *pub*,ID and *sk*_ID_ are private “member variables” of *𝓐*.

**Algorithm** *𝓐*.*OS*(*M*)

Begin

$\begin{array}{cc} output & Sign_{sk_{\text{ID}}}\left(M\right) \end{array}$

End

**Algorithm** *𝓐*.*OS*(*id*)

Begin

 IF(ID = *id*) *output* ⊥

 Else *output Enroll*(*pub*,*M*,*id*)

End

**Algorithm** *𝓑*.CipherTextGen(challenge)

Begin

$\left(sk,sk',n,\hat{e},G,G_{T},g\right)\leftarrow challenge$

$\left(PK_{e},SK_{e}\right)\leftarrow AsymEnc.KGen\left(n,\hat{e},G,G_{T},g\right)$

$d\overset{\$}{\leftarrow }\left\{0,1\right\}$

$x_{1},y_{1}\overset{\$}{\leftarrow }Z_{n},x_{2},y_{2}\overset{\$}{\leftarrow }Z_{n},x_{3},y_{3}\overset{\$}{\leftarrow }Z_{n}$

$C_{x_{1}}\leftarrow g^{ax_{1}},C_{y_{1}}\leftarrow g^{by_{1}}$

$C_{x_{2}}\leftarrow g^{ax_{2}},C_{y_{2}}\leftarrow g^{by_{2}}$

$C_{x_{3}}\leftarrow g^{ax_{3}},C_{y_{3}}\leftarrow g^{by_{3}}$

 IF(*d* = 0)

  (*K*_1_,*K*_2_,*K*_3_)←*sk*

 Else

  (*K*_1_,*K*_2_,*K*_3_)←*sk*′

$\tilde{K_{1}}\leftarrow g^{x_{1}+y_{1}}\cdot K_{\text{1}}{,}_{}\tilde{K_{2}}\leftarrow g^{x_{2}+y_{2}}\cdot K_{2}{,}_{}\tilde{K_{3}}\leftarrow g^{x_{3}+y_{3}}\cdot K_{3}$

$ct\leftarrow \left(C_{x_{1}},C_{y_{1}},C_{x_{2}},C_{y_{2}},C_{x_{3}},C_{y_{3}},\tilde{K_{1}},\tilde{K_{\text{2}}},\tilde{K_{\text{3}}}\right)$

*output ct,PK_e_*

End

**Algorithm** *𝓐.Guess*(*ct,st*)

Begin

$\left(\left(n,\hat{e},G,G_{T},PP\right),\left(MK,TK\right)\right)=\left(pub,priv\right)\leftarrow st$

$\text{Generate }C_{pub,sk_{\text{ID}},PK_{e}}\text{ according to the EGS functionality;}$

$\text{Generate }R_{pub,ct,PK_{e}}\text{ that works the same as }R_{pub,z,PK_{e}}{\text{ that is generated by Obf}}_{EGS};$

$\text{IF}\left(1={𝓓}^{\langle \langle C_{pub,sk_{\text{ID}},PK_{e}}\left(\right),Sign_{sk_{\text{ID}}}\left(\right)\rangle \rangle }\left(R_{pub,ct,PK_{e}}\right)\right)$

*d*' ← 0

 Else

$d'\overset{\$}{\leftarrow }\left\{0,1\right\}$

*output d'*

End

Note that if *d* = 1, $R_{pub,ct,PK_{e}}$ is the same as $R_{pub,Junk,PK_{e}}()$ which is generated by *Sim*, otherwise, $R_{pub,ct,PK_{e}}$ is really a valid output of Obf_*EGS*_.

**Algorithm** 𝓐.*OC*(*M*)

Begin

$//\text{Compute }EGS_{pub,sk_{\text{ID}},PK_{e}}(M)$

$\begin{array}{cc} output & C_{pub,sk_{\text{ID}},PK_{e}}(M) \end{array}$

End

We compute Pr[*d*′ = 0∧*d* = 0] and Pr[*d*′ = 1∧*d* = 1] in the security game as follows.

Pr[d'=0∧d=0]=Pr[d=0]⋅Pr[1←D<<Cpub,skID,PKe(),SignskID(),EnrollMK[id≠ID]()>>(Rpub,ct,PKe)|d=0]+Pr[d=0]⋅(12⋅Pr[1←D<<Cpub,skID,PKe(),SignskID(),EnrollMK[id≠ID]()>>(Rpub,ct,PKe)|d=0])=12⋅PrNice+12⋅(12⋅(1−PrNice))=PrNice+14 (27)

Pr[d'=1∧d=1]=Pr[d=1]⋅(12⋅Pr[1←D<<Cpub,skID,PKe(),SignskID(),EnrollMK[id≠ID]()>>(Rpub,ct,PKe)|d=1])=12⋅12⋅(1−PrJunk)=1−PrJunk4 (28)

Finally, the advantage of *𝓐* (as an adversary in a chosen-plaintext attack against the linear encryption scheme *LE*) can be calculated as follows.

$$\begin{array}{l} Adv_{𝓐}^{IND-CPA,LE}\\ =2\cdot \text{Pr}\left[d'=d\right]-1=2\cdot \left(\text{Pr}\left[d'=0\wedge d=0\right]+\text{Pr}\left[d'=1\wedge d=1\right]\right)-1\\ =2\cdot \left(\frac{{\text{Pr}}_{Nice}+1}{4}+\frac{1-{\text{Pr}}_{Junk}}{4}\right)-1=2\cdot \left(\frac{1}{2}+\frac{{\text{Pr}}_{Nice}-{\text{Pr}}_{Junk}}{4}\right)-1=\frac{{\text{Pr}}_{Nice}-{\text{Pr}}_{Junk}}{4}\\ =\frac{\delta }{2} \end{array}$$ (29)

Recall that *δ* = Pr_*Nick*_-Pr_*Junk*_, if *δ* is non-negligible, so is $Adv_{𝓐}^{IND-CPA,LE}$. This contradicts the security property (i.e., semantically secure against chosen-plaintext attacks) of the linear encryption scheme based on the decisional linear assumption. This ends the proof.

#### Remark 8

By a natural extension on the proof of the security of the ElGamal encryption scheme, the linear encryption scheme is semantically secure against chosen-plaintext attacks, assuming the decisional linear assumption holds [46].

#### Remark 9

Definition 3 (ACVBP w.r.t. Dependent Oracles and Restricted Dependent Oracles) fits for many application scenarios. Examples are shown in Table 6. Moreover, the proof of Theorem 3 does not work under the ACVBP or ACVBP w.r.t. Dependent Oracles.

**Table 6:** Some of the scenarios that Definition 3 should be used.

Cryptosystem	Restricted oracle
Identity-based cryptosystem	Extract the private key of an identity *id*≠*ID*
Forward-secure cryptosystem	Get the private key of a time period *t*′<*t*
*t*, *N*-key-insulated cryptosystem	Get the user keys of at most *t* time periods
*t*, *N*-threshold cryptosystem	Get at most *t*-1 pieces of the shared secret

#### 4.3.3. FT and FA w.r.t. the proposed EGS Obfuscator

We prove the relationship between Definition 3, 4, 6 and 8 which is already shown in Fig 7 as follows.

**Theorem 3**. If an obfuscator Obf_*EGS*_ for a EGS functionality satisfies RR&ACVBP w.r.t. dependent oracle $T\left(C\right)=Sign_{sk_{\text{ID}}}$ and restricted dependent oracle $𝓡(C)=Enroll_{MK}^{\left[id\neq \text{ID}\right]}$, then the FT w.r.t. EGS Functionality implies the FT w.r.t. EGS Obfuscator.

**Proof**. Suppose that a group signature scheme is FT w.r.t. the EGS functionality but not FT w.r.t. Obf_*EGS*_. There exists a PPT oracle machine *𝓕* (forgery) and *Q*_Obf_ ∈ ℕ such that $Adv_{𝓕}^{\left(Q_{\text{Obf}}\right)}=\text{Pr}\left[Exp_{EGS,{\text{Obf}}_{EGS},𝓕}^{Trace}\left(\lambda ,k,m\right)\right]$ is not a negligible value where the superscript *Q*_Obf_ implies that *𝓕* queries the oracle O_*EGS*_(·) at most *Q*_Obf_ times.

We denote the maximum advantage of the forgery *𝓕* which can query the oracle O_*EGS*_(·) at most *q*_Obf_(1≤*q*_Obf_≤*Q*_Obf_) times in the experiment $Exp_{EGS,{\text{Obf}}_{EGS},𝓕}^{Trace}(\lambda ,k,m)$ as $Adv_{𝓕}^{\left(q_{\text{Obf}}\right)}$. Clearly, we have

$$Adv_{𝓕}^{\left(Q_{\text{Obf}}\right)}\geq Adv_{𝓕}^{\left(Q_{\text{Obf}}-1\right)}\geq \cdots \geq Adv_{𝓕}^{1}\geq 0$$ (30)

Therefore, ∃*q′*_Obf_(1≤*q′*_Obf_≤*Q*_Obf_), s.t.

$$Adv_{𝓕}^{\left(q{'}_{\text{Obf}}\right)}-Adv_{𝓕}^{\left(q{'}_{\text{Obf}}-1\right)}\geq \frac{Adv_{𝓕}^{\left(Q_{\text{Obf}}\right)}}{Q_{\text{Obf}}}$$ (31)

We design the experiment $Exp_{{\text{Obf}}_{EGS},\text{ID}}^{Nice\_or\_Junk}$ as follows. In the experiment, the simulator *Sim* works the same as in the proof of Theorem 2.

$Exp_{{\text{Obf}}_{EGS},\text{ID}}^{Nice\_or\_Junk}$

Begin

$\left(pub,priv\right)=\left(\left(n,\hat{e},G,G_{T},PP\right),\left(MK,TK\right)\right)\leftarrow Setup\left(1^{\lambda },1^{k},1^{m}\right)$

$\left(PK_{e},SK_{e}\right)\overset{\$}{\leftarrow }EKGen\left(pub\right)$

$sk_{\text{ID}}=\left(K_{1},K_{2},K_{3}\right)\overset{\$}{\leftarrow }Enroll\left(pub,MK,\text{ID}\right)$

$C^{\left(0\right)}\leftarrow Sim^{\langle \langle C_{pub,sk_{\text{ID}},PK_{e}}\left(\right)\rangle \rangle }\left(\right);C^{\left(1\right)}\leftarrow {\text{Obf}}_{EGS}\left(C_{pub,sk_{\text{ID}},PK_{e}}\right)$

$coin\overset{\$}{\leftarrow }\left\{0,1\right\}$

C* = *C* ^(*coin*)^

$b\leftarrow {𝓓}^{\langle \langle C_{pub,sk_{\text{ID}},PK_{e}}\left(\right),Sign_{sk_{\text{ID}}}\left(\right),Enroll_{MK}^{\left[id\neq \text{ID}\right]}\left(\right)\rangle \rangle }\left(C*\right)$

End

Furthermore, we design a security game as follows.

Game2ExpEGS,𝓕Trace↔ExpObfEGS,IDNice_or_Junk↔𝓕→return a bit b𝓓output bSystem

We list the usage of the algorithms of *𝓓* that are used in Game2, i.e., *𝓓.Init*(), *𝓓.Sig*(*id*, *M*), *𝓓.Answer_O*_*EGS*_(*id*) and *𝓓.RR*, in Table 7.

**Table 7:** The algorithms of *𝓓* in the Game2.

Algorithm	Usage
*𝓓.Init*()	Initiate the values of private keys except for *id* = *ID*.
*𝓓.Answer_O*_*EGS*_(*id*)	Reply the $O^{<<O_{EGS}\text{(}id\text{)}>>}$ queries from the forgery *𝓕*.
*𝓓.Sig*(*id*, *M*)	Reply the $O^{<<Sign_{sk_{\text{ID}}}\left(\right)>>}$ queries from the forgery *𝓕*.
*𝓓.RR*(*C*)	Re-randomize the input obfuscated circuit *C*.

The existence of *𝓓.RR* is guaranteed by the hypothesis. The other three algorithms work as follows.

**Algorithm** *𝓓.Init*( )

Begin

 While(*id*<2^*k*^) do

  IF (*id* ≠ ID)

$SK[id]\leftarrow Enroll_{MK}^{\left[id\neq \text{ID}\right]}\left(id\right)$

*id* ← *id* + 1

 EndWhile

End

**Algorithm** *𝓓*.*Sig*(*id*, *M*)

Begin

 IF (*id* = *ID*)

$output\;Sign_{sk_{\text{ID}}}(M)$ //Get the output via oracle query

 Else

*output Sign*_*sk[id]*_ (*M*) //Compute the output directly

End

**Algorithm** *𝓓*.Answer_O_EGS_(id)

Begin

 IF (id = ID)

*𝓓*.RR(C*)

 Else

$output{\text{ Obf}}_{EGS}(C_{pub,SK[id],PK_{e}})$

End

Let $Adv_{𝓕}^{\left(q{'}_{\text{Obf}}\right)-Junk}$ be the probability of *𝓕* (and *𝓓*) outputs 1 when *coin* = 0 and *𝓕* queries $O^{<<O_{EGS}\text{(}id\text{)}>>}$ at most *q*′_Obf_ times. Clearly, we have

$$Adv_{𝓕}^{\left(q{'}_{\text{Obf}}\right)-Junk}\leq Adv_{𝓕}^{\left(q{'}_{\text{Obf}}-1\right)}$$ (32)

Let $Adv_{𝓕}^{\left(q{'}_{\text{Obf}}\right)-Nick}$ be the probability of *𝓕* (and *𝓓*) outputs 1 when *coin* = 1 and *𝓕* queries $O^{<<O_{EGS}\text{(}id\text{)}>>}$ at most *q*′_Obf_ times. $Adv_{𝓕}^{\left(q{'}_{\text{Obf}}\right)-Nick}$ equals to $Adv_{𝓕}^{\left(q{'}_{\text{Obf}}\right)}$.

Hence, *𝓓* is a distinguisher of a simulator and a real obfuscator because:

$$Adv_{𝓓}^{\left(q{'}_{\text{Obf}}\right)}≜\left|Adv_{𝓕}^{\left(q{'}_{\text{Obf}}\right)-Nice}-Adv_{𝓕}^{\left(q{'}_{\text{Obf}}\right)-Junk}\right|\geq Adv_{𝓕}^{\left(q{'}_{\text{Obf}}\right)}-Adv_{𝓕}^{\left(q{'}_{\text{Obf}}-1\right)}\geq \frac{Adv_{𝓕}^{\left(Q_{\text{Obf}}\right)}}{Q_{\text{Obf}}}$$ (33)

In the above analysis, we show that, if the FT w.r.t. EGS Functionality is satisfied, but the FT w.r.t. EGS Obfuscator is NOT satisfied, then it contradicts the RR&ACVBP w.r.t. dependent oracle $T\left(C\right)=Sign_{sk_{\text{ID}}}$ and restricted dependent oracle $𝓡(C)=Enroll_{MK}^{\left[id\neq \text{ID}\right]}$. This ends the proof.

The following propositions are easy to verify, hence we omit the proofs.

#### Proposition 1

The proposed obfuscator Obf_*EGS*_ is rerandomizable w.r.t. the dependent oracle $T\left(C\right)=Sign_{sk_{\text{ID}}}$ and the restricted dependent oracle $𝓡(C)=Enroll_{MK}^{\left[id\neq \text{ID}\right]}$.

#### Proposition 2

The group signature scheme in [45] satisfies the following security properties with regard to the proposed EGS functionality or the proposed obfuscator Obf_*EGS*_:

1. FT w.r.t. the proposed EGS functionality.
2. FA w.r.t. the proposed EGS functionality.
3. FT w.r.t. the proposed obfuscator Obf_*EGS*_.
4. FA w.r.t. the proposed obfuscator Obf_*EGS*_.

## Related Studies and Comparison

The work of Barak et al. [30] initiated the theoretical investigation of obfuscators and has been a landmark in the research of obfuscation. The main result is that **general-purpose** obfuscation is impossible even under rather weak security definitions. This result is extended in many publications, such as the impossibility of obfuscation with auxiliary input [32], the impossibility of approximate obfuscation [31], the impossibility of efficient best-possible obfuscation [33], and the impossibility of restricted circuit classes [29]. Because of the difficulties or even impossibilities of various obfuscations, it is challenging to find a secure obfuscator even for a special functionality.

Fortunately, some positive results were obtained besides these negative results. It was shown by Canetti that under a very strong Diffie-Hellman assumption, point functions can be obfuscated [51]. Further work from Wee [52] relaxes the assumptions required for obfuscation. Lynn et al. [53] gave several provable obfuscations for complex access control functionalities in the random oracle model. Moreover, Hofheinz et al. [42] provided some specific examples also with theoretical importance. One example is that we can easily transform an asymmetric encryption scheme into an obfuscatable symmetric encryption scheme, another example is that we can easily transform digital signature scheme into an obfuscatable MAC (Message Authentication Code).

However, these positive results mainly serve as theoretical illustrations. For instance, because the speed of the encryption algorithm in an asymmetric encryption scheme is usually much slower than that of a traditional block cipher, the strongly obfuscatable symmetric encryption scheme [42] is not suitable for practice. It is similar for the obfuscatable MAC [42], because the speed of verification algorithm in a digital signature scheme is usually much slower than that of using a keyed-Hash Message Authentication Code (HMAC).

Besides these positive results for theoretical study, some obfuscatable cryptographic functionalities and corresponding obfuscators for these cryptographic functionalities with acceptable runtime costs are introduced in recent years. In Table 8, we list the functionalities and obfuscators to the extent of our knowledge, and the last line is the proposed scheme in this paper. Furthermore, we provide a comparison on the security notions of obfuscation for different signature-related schemes in Table 9.

**Table 8:** A comparison of highly relative studies.

Functionality	Year	Base scheme(s) or building component(s)	Complexity Assumptions
Re-encryption [35]	2007	The linear encryption scheme (in [46]).	DLIN
Encrypted signature [34]	2010	The linear encryption scheme (in [46]), and Water’s signature scheme (in [54]).	DBDH, DLIN
Encrypted verifiable Encrypted signature [36]	2011	The linear encryption scheme (in [46]), and Water’s signature scheme (in [54]).	Exponent l-weak DH, DBDH, DLIN
Two-step oblivious signature [38]	2012	The linear encryption scheme (in [46]), Water’s signature scheme (in [54]), and Pedersen’s VSS protocol (in [55]).	SDHI, DLIN
Functional re-encryption [37]	2012	Re-Encryption (in [35])	SXDH
Encrypted Blind Signature [49]	2013	Schnorr’s Blind Signature, and the linear encryption scheme (in [46]).	DH
Encrypted Proxy Signatures [50]	2013	Tightly Structure-Preserving Signatures (in [56]), and the linear encryption scheme (in [46]).	DLIN, DBDH
Conditional Re-encryption with Keyword Search [39]	2013	A modified version of ElGamal encryption.	DBDH
Encrypted Verifiably Encrypted Signatures [41]	2014	The linear encryption scheme (in [46]), and Water’s signature scheme (in [54]).	CDH, AgExt, DLIN
Re-encryption, Functional Re-encryption, and Multi-hop Re-encryption [40]	2014	Regev’s encryption scheme (in [57])	DLWE
Encrypted Group Signature (**this paper**)		The linear encryption scheme (in [46]), and a group signature scheme (in [45]).	CDH, SD, HSDH, DLIN

Acronyms used in the last column are explained as follows:

• Decisional Linear (DLIN);

• Decisional Bilinear Diffie-Hellman (DBDH);

• Strong Diffie Hellman Indistingshuishability (SDHI);

• Symmetric External Diffie-Hellman (SXDH);

• Diffie-Hellman (DH);

• Computational Diffie-Hellman (CDH);

• Aggregate Extraction (AgExt);

• Decisional Learning with Errors (DLWE);

• Subgroup Decision (SD);

• Hidden Strong Diffie-Hellman (HSDH).

**Table 9:** A comparison on the security notions of obfuscation for different signature-related schemes.

Reference No.	The main Security notion for the obfuscator	The dependent oracle(s)	The restricted dependent oracle(s)	The scheme-related security notion(s) w.r.t Obfuscator
[34]	ACVBP w.r.t. DOs	Sign	N/A	EU w.r.t. ES Functionality
[36]	ACVBP w.r.t. DOs	Sign	N/A	/
[38]	ACVBP w.r.t. DOs	Sign	N/A	/
[49]	ACVBP w.r.t. DOs	Sign	N/A	Blindness w.r.t. EBS Obfuscator; One-more Unforgeability w.r.t. EBS Obfuscator
[50]	ACVBP w.r.t. DOs	Sign	N/A	EU w.r.t. ES Functionality
[41]	ACVBP w.r.t. DOs	Sign	N/A	EU w.r.t. EVES Obfuscator; Opacity w.r.t. EVES Obfuscator
This paper	ACVBP w.r.t. DOs **and RDOs**	Sign	Enroll	FT w.r.t. EGS Obfuscator; FA w.r.t. EGS Obfuscator

Acronyms used in the above table are explained as follows

• Dependent Oracle (DO)

• Restricted Dependent Oracle (RDO)

• Existential Unforgeability (EU)

• Encrypted Blind Signature (EBS)

• Encrypted Verifiably Encrypted Signatures (EVES)

From Table 8, we can see that the proposed obfuscator is the first obfuscator for group oriented security schemes. Furthermore, as shown in Table 9, ACVBP w.r.t. DOs **and RDOs** introduced in this paper is the first security notion to fulfill the security requirement to protect a signature related scheme against collusion attacks. This new security notion should also be used to capture the security requirements of obfuscation of identity-based cryptosystems, forward-secure cryptosystem, key-insulated cryptosystem and threshold cryptosystem.

As we have mentioned at the end of section 2.1, there are two general-purpose obfuscators (in [43,44,58]) proposed in 2013 and 2014.

The first one is constructed for indistinguishability obfuscation that supports all polynomial-size circuits, which were given by Garg et al. [43] and strengthened by Barak et al. [58]. However, it uses a weak security notion of obfuscation, i.e. the indistinguishability obfuscation [29] which says that, for any pair of circuits *C*_0_ and *C*_1_ that agree on all inputs *C*_0_(*x*) = *C*_1_(*x*), it should be hard to distinguish the obfuscation of *C*_0_ from that of *C*_1_. The new security notion used in this paper, i.e. the ACVBP w.r.t. DOs and RDOs, is stronger than the indistinguishability obfuscation.

The second general-purpose obfuscator is capable of obfuscating all polynomial size circuits [44]. The obfuscator, which uses graded encoding schemes is proven that the obfuscator exposes no more information than the program’s black-box functionality, and achieves virtual black-box security, in the generic graded encoded scheme model. The obfuscator is obtained by developing techniques used to obfuscate d-CNF formulas in [59], and applying them to permutation branching programs. This yields an obfuscator for circuits in the complexity class NC1 and the obfuscator can be extended to a more powerful one for any polynomial-size circuit by using the homomorphic encryption technique. However, the complexity and expansion rate of homomorphic encryption are too large to be applied in practical applications.

### Remark 10

NC1 denotes the class of decision problems decidable by uniform boolean circuits with a polynomial number of gates of at most two inputs and depth O(logn), or the class of decision problems solvable in time O(logn) on a parallel computer with a polynomial number of processors. Many cryptographic functionalities are out of this class.

### Remark 11

The impossibility results in [29,30,32] do not extend to idealized models, such as the random oracle model, the generic group model, and particularly the generic graded encoding model which is used in [44], hence the “generic purpose obfuscator” does not contradict these impossibility results.

Hence, although there have been some important advances in general-purpose obfuscation, such as in [43,44,58], there is no practically general approach for designing obfuscators under the security notion used in this paper. Therefore, it is a challenging work to find an obfuscatable encrypted group signature (EGS) functionality and design a corresponding efficient obfuscator.

## Discussions

In this section, we first introduce possible applications and extensions of the proposed technique. Then, the rationale behind the obfuscatable sign-then-encrypt functionalities are investigated. Finally, the contribution of our findings is discussed at the last subsection.

### 6.1. Possible Applications and Extensions

Group signature schemes are applicable in many practical applications, such as in social networks [3,4], medical information systems [5–7], VANets [8,9], electronic voting [10], WSNs [11], electronic cash [12,13], and cloud computing [14–18]. These studies make great contributions for protecting security of information systems and privacy of users against various attacks. However, these applications are rather complicated. Therefore, to illustrate the applicability of the proposed technique, two simple examples are provided in section 6.1.1 and 6.1.2.

Moreover, the proposed technique can be adapted to identity-based cryptography and key-insulated cryptography. These extensions are introduced in section 6.1.3.

#### 6.1.1. An Application in cloud computing

**Fig 8:**
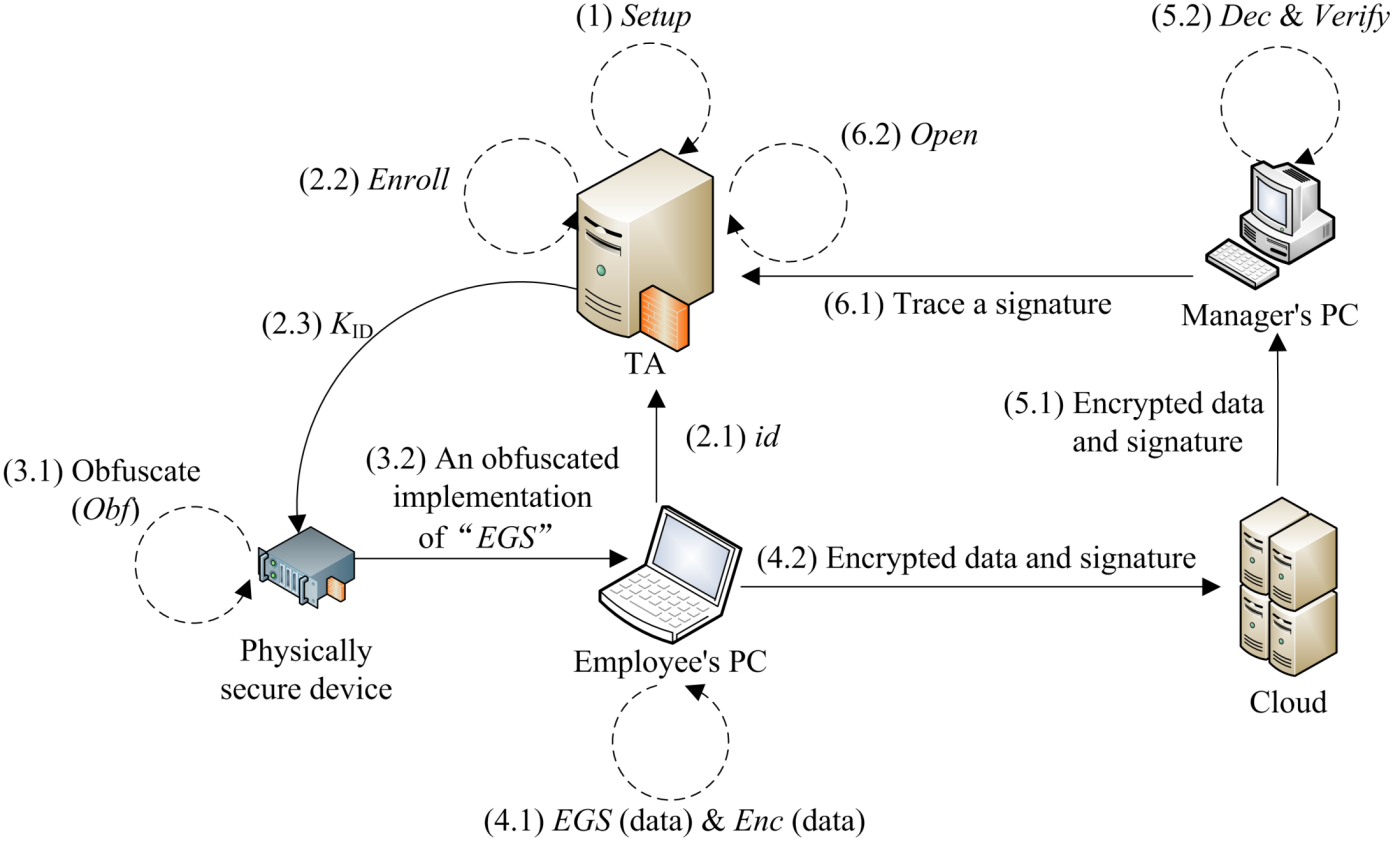
An application in cloud computing.

Group signature technique is perfectly suited for privacy-preserving security schemes in cloud computing. The proposed application is inspired by [18]. The application scenario for encrypted group signature schemes is a single company hosting a private cloud for its employees, e.g. providing access to file sharing or printing services. Usually, there is no need to identify the employee who has uploaded a certain file, and in some cases this may even be a confidential information (e.g. for labor unions within the company). However, in the event of uploading a file illegally, the company’s management may have a severe interest in finding the responsible employee, regardless of potential reasons for preserving anonymity. The application is illustrated in Fig 8.

#### 6.1.2. An Application in mobile healthcare social networks

The second application is a privacy-preserving emergency call scheme for mobile healthcare social networks. It is adapted from [4].

**Fig 9:**
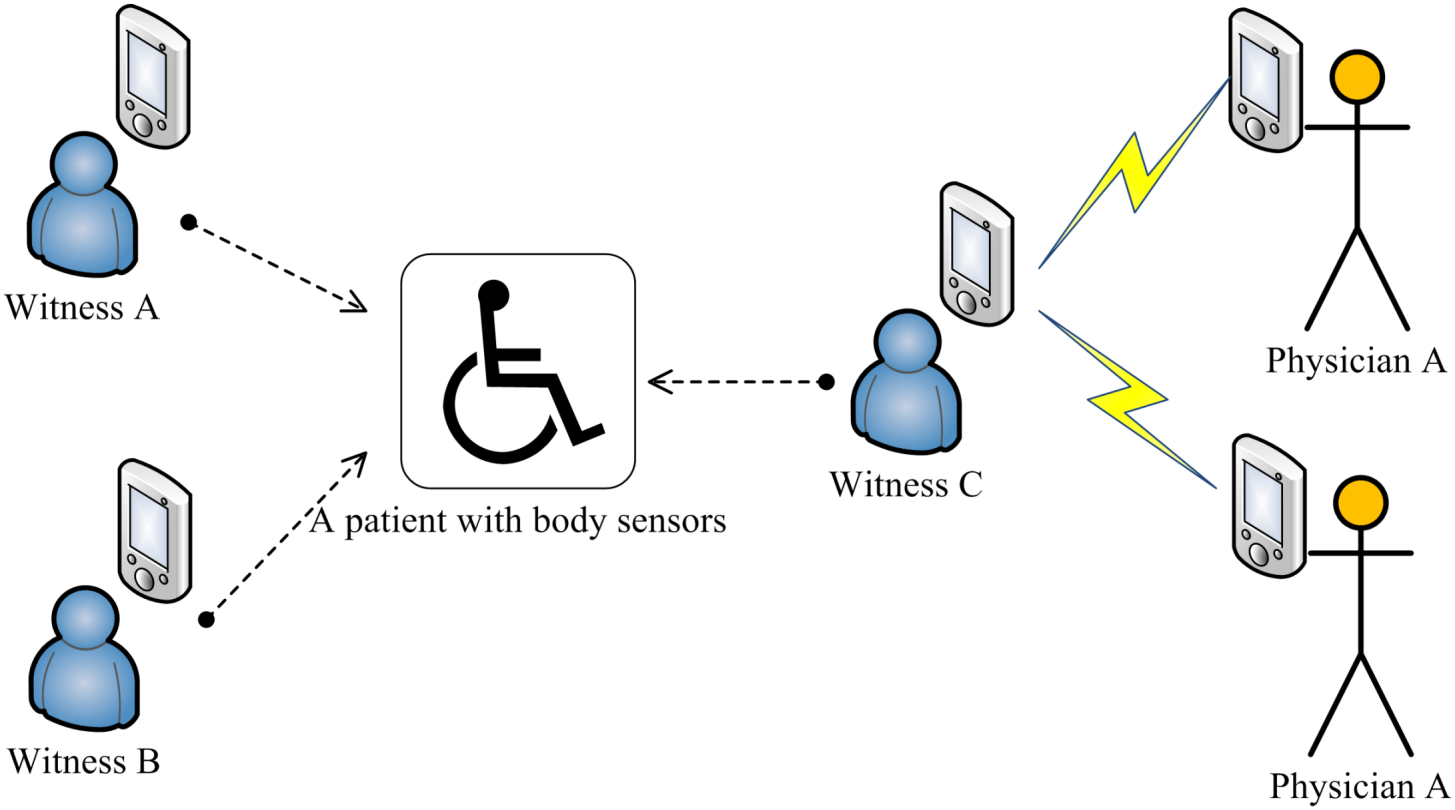
A decentralized emergency response scheme.

As illustrated in Fig 9, a privacy-preserving emergency call system enables patients in life-threatening emergencies to accurately and fast transmit emergency data to the nearby helpers via mobile healthcare social networks. Once an emergency happens, the personal digital assistant (PDA) of the patient runs the emergency call procedure to collect the emergency data including patient health record, patient physiological condition, as well as the current emergency location. The emergency call procedure then generates an emergency call with the emergency data inside and epidemically disseminates it to every user in the patient’s neighborhood. If a physician happens to be nearby, the PEC ensures the time used to notify the physician of the emergency to be the shortest.

**Fig 10:**
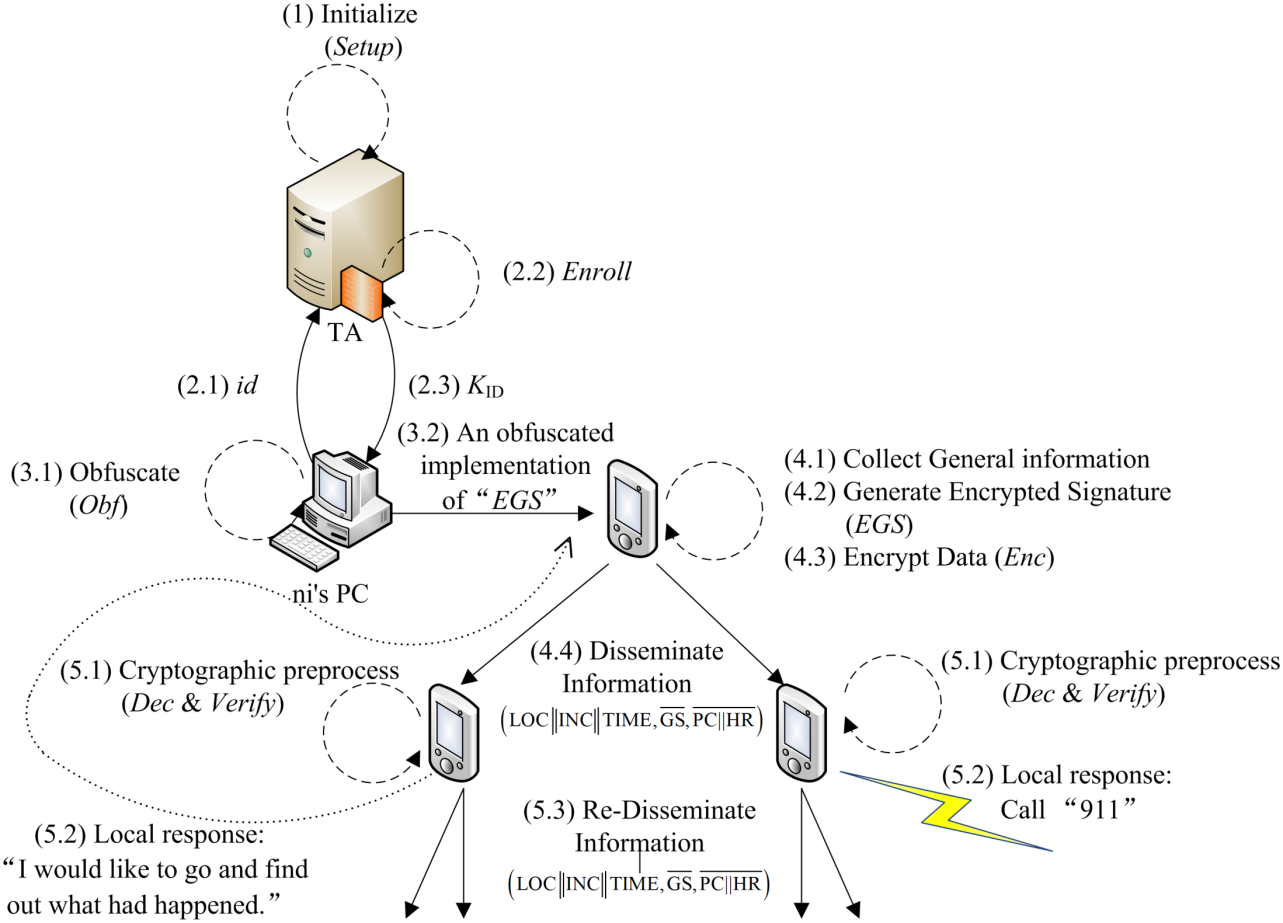
An application in a privacy-preserving emergency call system based on mobile social network.

In an emergency situation, a patient must reveal his/her information to the nearby users in order to ask for their instant help. However, with privacy concerns, the patients would preserve the identity privacy and prevent their transactions being linked to their unique identities. On the other hand, a TA must be able to trace the emergency call and identify the corresponding patient. In this way, any malicious attacker who has generated a bogus emergency call would be detected and punished. Details of the application are listed as follows and illustrated in Fig 10.

(1) Initialization Phase:

At the beginning, the Trusted Authority (TA) uses the algorithm *Setup* to generate system parameters, public values, the master enrollment key *MK*, and the group manager’s tracing key *TK*.

(2) Registration Phase:

When a patient who needs medical services for possible emergency situations joins the system, TA uses the algorithm *Enroll* to issue a secret signing key *sk* and delivers the key to the patient through a secure channel.

(3) Obfuscation phase:

The patient uses the obfuscator Obf_*EGS*_ to generate an obfuscated implementation of encrypted group signature functionality $EGS_{pub,sk,PK_{e}}$ in his/her personal computer. Then the patient transfers the obfuscated implementation to his/her PDA.

(4) Emergency call generation phase:

The emergency call generation is started by a detection of the abnormal physiological condition from body sensors. This condition can be pre-implanted into the patient’s PDA with the instructions of the medical professionals.

Let patient *n*_*i*_ denote a user who has an emergency situation. The patient *n*_*i*_’s PDA generates an emergency call according to the following steps.

(4.1) General information collection phase: The PDA intelligently collects the following general information:

- Location (LOC): It contains the emergency location information which can be measured by a global positioning system (GPS) of the patient PDA.
- Incident (INC): It contains a general description of the environment where the emergency occurs.
- Time (TIME): It contains the exact time when the emergency occurs.

(4.2) Encrypted Signature generation phase: The PDA uses the obfuscated implementation EGS to generate an encrypted group signature on “LOC||INC||TIME”. The encrypted group signature will be put into the “EGS” component.

(4.3) Data encryption phase: The PDA encrypts the patient *n*_*i*_’s physiological condition (PC) and health record (HR).

(4.4) Information dissemination phase: The PDA disseminates the Emergency Call Message $\text{ECM=LOC}\left|\left|\text{INC}\right|\right|\text{TIME},\bar{\text{GS}},\bar{\text{PC||HR}}$ to the neighboring users and APs (Access Points). Note that $\bar{\text{GS}},\bar{\text{PC||HR}}$ denotes the ciphertexts of GS and PC||HR where $\text{EGS=}\bar{\text{GS}}$.

(5) Emergency call response phase:

User *n*_*j*_ receives the ECM from patient *n*_*i*_ and executes the following steps, where a PDA represents user *n*_*j*_ ‘s PDA:

(5.1) Cryptographic preprocess: First, the PDA decrypts the encrypted part of the message ($\bar{\text{GS}},\bar{\text{PC||HR}}$) and gets the plaintext GS,PC||HR. Second, the PDA verifies the group signature “GS” by using the verification algorithm *Verify*. If the verification passes, user *n*_*j*_ confirms the information “LOC*||*INC*||*TIME.”

(5.2) Local response: As an emergency response, user *n*_*j*_ firstly makes a phone call (e.g., dialing 911) to report the emergency to the hospital/first-aid center. Then, the PDA executes the step (5.3).

(5.3) Information re-dissemination phase: The PDA checks the “TIME” component: If the time period from the emergency occurrence to the user *n*_*j*_ receiving it, is larger than the threshold value, then the emergency call is discarded. Otherwise, the PDA forwards the ECM to the neighboring users.

#### 6.1.3. Extensions to identity-based signatures and key-insulated signatures

In a traditional (certification-based) public-key cryptosystem, the association between a user’s identity and his/her public key is obtained through a digital certificate issued by a Certifying Authority (CA). The CA checks the credentials of a user before issuing a certificate to the user. If a signer wants to sign a message, first the signer obtains a digital certificate for his/her public key from a CA. The signer then signs the message using the private signing key and sends the signed message along with his/her certificate to the receiver. The receiver (verifier) first verifies the validity of the certificate by checking the certificate revocation list published by the CA, then the receiver verifies the signature using the public key in the certificate. If many CAs are involved between the signer and the verifier, then the entire certificate path has to be verified.

Hence, the process of certificate management requires high computational and storage efforts. To simplify the certificate management process, Shamir [60] introduced the concept of identity-based cryptosystem. In such cryptosystems the public key of a user is derived from his/her identity information and a private key is generated by a Trusted Authority (TA). The advantage of an identity-based cryptosystem is that it simplifies the key management process which is a heavy burden in the traditional certificate based cryptosystems. In these cryptosystems, the verifier can verify the signer’s signature just by using his/her identity information. In general, an identity based cryptosystem has the following properties:

- user’s public key is his/her identity (or derived from the identity).
- no requirement of public key directories
- the verification process of a signature requires only the signers’ identity (along with some public system parameters)

These properties make identity-based cryptosystems advantageous over the traditional certification-based cryptosystems, as key distribution is far simplified. It needs a directory only for authenticated public system parameters of the KGC (Key Generation Center), which is clearly less burdensome than maintaining a public key directory for total users.

As a by-product of the paper, we found that one could easily transform the proposed functionality to an obfuscatable encrypted identity-based signature by applying the following steps.

1. The KGC sets up the group for all users.
2. The KGC broadcasts the tracing key as a public value.
3. When generating a signature, the signer appends his/her identity at the end of the signature.
4. Merge the verification algorithm and the opening algorithm together.
5. The obfuscators, and even the encryption key generation algorithm, the encryption algorithm and the decryption algorithm can be used in the identity-based scenario without modification.

Note that the main security definition (ACVBP w.r.t. DOs and RDOs) can be almost directly obtained from Section 4. Moreover, the security proof can be deducted from this paper easily. So we omit the details in this paper.

Furthermore, as suggested in [61], by identifying time periods with identities, any identity-based signature scheme yields a perfectly (but not necessarily strong) key-insulated signature scheme. Accordingly, by applying this technique to the above identity-based scheme, an obfuscatable key-insulated signature scheme and a corresponding obfuscator is acquired.

Hence, besides the encrypted group signature, this paper adds two more new items in the list of obfuscatable cryptographic functionalities, i.e., the encrypted identity-based signature and the encrypted key-insulated signature.

### 6.2. The Rationale Behind the Obfuscatable Sign-Then-Encrypt Functionalities

Intuitively, design a sign-then-encrypt functionality needs a signing algorithm and an asymmetric encryption algorithm which are “commutable”. Hence, the obfuscator can encrypt the private signing key by using the encryption algorithm with the public encryption key. In fact, all the works which are listed in Table 9 follow the thread of the idea in [34]. We explain the idea formally as follows.

In a digital signature scheme, the signing algorithm *𝓢* takes the secret signing key *sk* ∈ *𝕊*_*SSk*_ and a message *M* ∈ *𝕊*_*Msg*_ to return a signature (also sometimes called a tag) *σ* ∈ {0,1}*∪⊥. The algorithm may be randomized. We write *𝓢*(*sk*, *M)* for the operation of running Sign on inputs *sk*, *M* and letting *σ* be the signature returned.

In an asymmetric encryption scheme, the encryption algorithm *𝓔* takes the public encryption key *PK* ∈ *𝕊*_*PEK*_ and a plaintext *M* ∈ *𝕊*_*PT*_ to return a value called the ciphertext. The algorithm may be randomized. We write *C*←*𝓔*(*PK*, *M*) for the operation of running *𝓔* on inputs *PK*, *M* and letting *C* ∈ *𝕊*_*CT*_ be the ciphertext returned.

Suppose the encryption is semantic secure (IND-CPA). If (34) holds, it is expected that we can obtain a secure obfuscator of *𝓔*∘*𝓢*.

$$\forall sk\in S_{SSK},\forall PK\in S_{PEK},\forall M\in S_{Msg},𝓔\left(PK,𝓢\left(sk,M\right)\right)=𝓢\left(𝓔\left(PK,sk\right),M\right)$$ (34)

Eq (34) implies that

$$𝓔\left(S_{PEK},S_{SSK}\right)\subseteq S_{SSK}$$

Usually, the signing algorithm and the encryption algorithm are randomized. Therefore, (34) could be relaxed. Consider the implicit usage of random variables in the encryption algorithm and signing algorithm, we denote the set of random variables in the encryption process as *R*_*E*_ and the set of random variables in the signing process as *R*_*S*_. Let ${𝓔}_{R_{E}}$ and ${𝓢}_{R_{S}}$ be the corresponding encryption process and signing process, respectively. In order to satisfy the requirement of preserving functionality, (35) is sufficient.

∀sk∈SSSK,∀PK∈SPEK,∀M∈SMsg,∃(RE,RE',RS,RS',),s.t.𝓔RE(PK,𝓢RS(sk,M))=𝓢RS'(𝓔RE'(PK,sk),M) (35)

As to the security requirement, the formal security analysis hinges on the security model of the signature scheme. Because there are many variations of digital signature schemes (e.g., group-oriented, identity-based, etc.), it is out of the scope of this paper.

### 6.3. The Contribution

The paper has introduced a new secure obfuscator for encrypted group signature and corresponding security notions.

Theoretically, six new security notions of the encrypted group signature functionality and its obfuscators are proposed. The notions are ACVBP w.r.t. w.r.t. Dependent Oracles (DOs) and Restricted Dependent Oracles (RDOs), rerandomizable w.r.t. dependent oracle set and restricted dependent oracle set, full-traceability w.r.t. EGS Functionality, full-anonymity w.r.t. EGS Functionality, full-traceability w.r.t. EGS Obfuscator, and full-anonymity w.r.t. EGS Obfuscator.

The most important one of the new security notions is ACVBP w.r.t. DOs and RDOs which describes the security requirement of protecting the output of the proposed obfuscator, i.e., the obfuscated implementation of encrypted group signature functionality against collision attacks from group members. The security notions fit for many other cryptographic schemes which collision attacks from users need to be considered, such as identity-based signature schemes, ring signature schemes, attribute-based signature schemes and key-insulated signature schemes.

Practically, as it was discussed in Section 5, a generic obfuscator (for various sign then encrypt functionalities) is hard to find. Therefore, the obfuscators for various sign then encrypt functionalities must be studied one by one. In [34], Hada proposed the first obfuscatable sign-then-encrypt functionality and a corresponding obfuscator. Since then, most of the research work has been done on proposing various sign-then-encrypt functionalities and obfuscators, such as obfuscators for oblivious signature, encrypted blind signature, encrypted proxy signature, and encrypted verifiably encrypted signature. Note that it is not a trivial work to find an obfuscatable cryptographic functionality. For example, many widely-used signature schemes have not been found any obfuscatable concrete scheme (even in the sign then encrypt form), such as identity-based signature schemes, attribute-based signature schemes and key-evolvement signature schemes (include forward-secure signature, key-insulated signature, and intrusion-resilient signature).

A special obfuscatable group signature functionality, i.e., the encrypted group signature, is proposed with a concrete scheme, and then a corresponding obfuscator is provided in this paper. The correctness and security of the proposed obfuscator are proven. Then the efficiency of the proposed encrypted group signature functionality and its obfuscator is analyzed. The results of this paper can be used as building blocks of privacy preserving security protocol of various emerging applications such as social networks, medical information systems, Vehicular Ad hoc Networks (VANets), electronic voting, Wireless Sensor Networks (WSNs), electronic cash, and especially cloud computing.

Finally, as by-products of this paper, besides the encrypted group signature, we add two more new items in the list of obfuscatable cryptographic functionalities, i.e., the encrypted identity-based signature and the encrypted key-insulated signature.

## Conclusions and Future Work

Group signature technique is used in many privacy-preserving security schemes for social networks, cloud computing, VANets, WSNs, electronic voting and electronic cash. To provide a building block for these schemes in white-box attack contexts, we give an obfuscatable EGS functionality, and then provide an obfuscator for the proposed EGS functionality. We also introduce a new security notion (ACVBP w.r.t. Dependent Oracles and Restricted Dependent Oracles) to capture the requirement of protecting the output of an obfuscator for EGS functionality against collision attacks from group members. Moreover, five other new security notions are also provided. We prove that the proposed obfuscator preserves EGS functionality and satisfies the proposed security notions. As a byproduct of this study, ACVBP w.r.t. Dependent Oracles and Restricted Dependent Oracles fits for many types of cryptosystems, such as identity-based cryptosystems, forward-secure cryptosystems, key-insulated cryptosystems and threshold cryptosystems. Finally, we have introduced two possible applications and two extensions of the proposed technique.

In the future, we plan to adopt the obfuscatable EGS functionality in practical security solutions for validation. By using the proposed obfuscatable EGS functionality as a building block, we also consider exploring novel approaches for designing privacy-preserving security schemes. Furthermore, we will try to explore the design strategy for generalized constructions of obfuscatable sign-then-encrypt functionalities.
